# Chronic Intermittent Ethanol Exposure Dysregulates Nucleus Basalis Magnocellularis Afferents in the Basolateral Amygdala

**DOI:** 10.1523/ENEURO.0164-22.2022

**Published:** 2022-11-10

**Authors:** Sarah E. Sizer, Michaela E. Price, Brian C. Parrish, Samuel H. Barth, Chelcie F. Heaney, Kimberly F. Raab-Graham, Brian A. McCool

**Affiliations:** Department of Physiology and Pharmacology, Piedmont Triad Community Research Center (PTCRC), Wake Forest University School of Medicine, Winston-Salem, NC 27101

**Keywords:** acetylcholine, amygdala, basal forebrain, ethanol, GABA, glutamate

## Abstract

Nucleus basalis magnocellularis (NBM) cholinergic projections to the basolateral amygdala (BLA) regulate the acquisition and consolidation of fear-like and anxiety-like behaviors. However, it is unclear whether the alterations in the NBM-BLA circuit promote negative affect during ethanol withdrawal (WD). Therefore, we performed *ex vivo* whole-cell patch-clamp electrophysiology in both the NBM and the BLA of male Sprague Dawley rats following 10 d of chronic intermittent ethanol (CIE) exposure and 24 h of WD. We found that CIE exposure and withdrawal enhanced the neuronal excitability of NBM putative “cholinergic” neurons. We subsequently used optogenetics to directly manipulate NBM terminal activity within the BLA and measure cholinergic modulation of glutamatergic afferents and BLA pyramidal neurons. Our findings indicate that CIE and withdrawal upregulate NBM cholinergic facilitation of glutamate release via activation of presynaptic nicotinic acetylcholine receptors (AChRs). Ethanol withdrawal-induced increases in NBM terminal activity also enhance BLA pyramidal neuron firing. Collectively, our results provide a novel characterization of the NBM-BLA circuit and suggest that CIE-dependent modifications to NBM afferents enhance BLA pyramidal neuron activity during ethanol withdrawal.

## Significance Statement

Chronic alcohol dysregulates the neural circuitry controlling behavioral responses to stress, emotion, and motivation, and produces maladaptive behaviors that cause relapse. Since nucleus basalis magnocellularis (NBM) cholinergic projections to the basolateral amygdala (BLA) regulate the acquisition/consolidation of fear and anxiety, we used electrophysiology to understand how alcohol withdrawal (WD) alters NBM neurons and measure downstream effects on their BLA projections. Our results provide a novel characterization of the NBM-BLA circuit and illustrate that alcohol withdrawal strengthens NBM cholinergic neurotransmission and upregulates glutamate signaling in the BLA through the activation of nicotinic acetylcholine receptors (AChRs). Collectively, these findings illustrate that modifications in NBM projections may disrupt the excitatory/inhibitory balance in the BLA and help promote BLA pyramidal neuron activity during alcohol withdrawal.

## Introduction

Alcohol use disorder (AUD) is a chronic brain disease characterized by cycles of excessive drinking, abstinence, and relapse. Chronic alcohol consumption causes structural and functional changes in stress and reward pathways that promote negative affect during abstinence (Koob and Volkow, 2016). Relapse is commonly motivated by a desire to alleviate the anxiety and craving associated with alcohol withdrawal (WD; Sliedrecht et al., 2019). Since anxiety during abstinence strongly correlates with the risk of relapse (Sliedrecht et al., 2019), understanding the neurophysiology of withdrawal-induced negative affect is essential to mitigate the incidence of relapse.

The basolateral amygdala (BLA) is a node of affective processing that regulates behavioral responses to emotionally salient stimuli. BLA pyramidal neurons receive sensory and cognitive-related information via glutamatergic afferents entering the region through distinct input pathways. One example is the stria terminalis (ST), a pathway that contains glutamatergic afferents from the medial prefrontal cortex and various thalamic nuclei (Ray and Price, 1992). Glutamate release from the ST drives BLA pyramidal neuron excitability and ultimately the activity of downstream efferent reward and aversion circuits. Chronic ethanol exposure and withdrawal increase glutamatergic signaling in the BLA, facilitate BLA pyramidal neuron excitability and ultimately the expression of anxiety-related and reward-related behaviors (Morales et al., 2018; McGinnis et al., 2020a,b; Price and McCool, 2022). The present study suggests that dysregulation of nucleus basalis magnocellularis (NBM) afferents potentiate BLA pyramidal neuron activity during withdrawal.

The NBM is part of the basal forebrain cholinergic system that projects throughout the cortex, hippocampus, and amygdala. Notably, the NBM is the primary source of cholinergic innervation in the BLA (Carlsen et al., 1985; Aitta-Aho et al., 2018); and, the projections are among the most dense cholinergic inputs in the CNS (Ben-Ari et al., 1977). Acetylcholine (ACh) released by NBM cholinergic projections binds to nicotinic and muscarinic ACh receptors (n/mAChRs) expressed by ST afferents, local GABAergic interneurons, and BLA pyramidal neurons (Zhu et al., 2005; Pidoplichko et al., 2013; Lee and Kim, 2019). NBM cholinergic input is thus poised to modulate BLA output by facilitating neurotransmitter release and fast synaptic transmission via ionotropic nAChRs and metabotropic mAChRs. NBM projections may also contain GABAergic and glutamatergic components (Gritti et al., 2006). One-third of NBM-BLA ChAT^+^ terminals express the vesicular glutamate transporter 3 (vGLUT3; Nickerson Poulin et al., 2006), which suggests a potential for the co-release of acetylcholine and glutamate from these synapses (Higley et al., 2011; Nelson et al., 2014). Additionally, ∼10% of NBM projections to the BLA are GABAergic (Mascagni and McDonald, 2009) and appear to synapse directly onto both BLA pyramidal neurons and local GABAergic interneurons (McDonald et al., 2011). However, the physiological significance of these NBM GABAergic projections and the potential for glutamate co-release from cholinergic terminals within BLA remains unclear.

Behavioral and electrophysiology studies illustrate that the NBM-BLA circuit facilitates learning by controlling the consolidation of salient cues. For example, NBM cholinergic neurons fire rapidly in response to appetitive or aversive stimuli (Zhang et al., 2004; Hangya et al., 2015; Rajebhosale et al., 2021) and cause transient and scaled increases in BLA acetylcholine levels (Mark et al., 1996; Jing et al., 2020; Liu et al., 2021). Optogenetic stimulation of ChAT^+^ terminal fields in the BLA enhances cue-reward learning (Crouse et al., 2020) and prolongs fear extinction (Jiang et al., 2016; Kellis et al., 2020). These preclinical data suggest that acetylcholine strengthens the acquisition of fear-associated and reward-associated memories by enhancing BLA pyramidal neuron activity. Electrophysiology studies support these findings and show that endogenous acetylcholine release increases *in vivo* and *ex vivo* neuron firing (Jiang et al., 2016), the signal-to-noise ratio (Unal et al., 2015), and elicits long-term potentiation (LTP; Jiang et al., 2016) in BLA pyramidal neurons. However, no studies have examined withdrawal-induced alterations in the NBM-BLA circuit and the subsequent effects on glutamatergic signaling in the BLA.

Previous findings suggest that chronic intermittent ethanol (CIE) exposure, a model that produces dependence and enhances anxiety-like behaviors in rodent models (Morales et al., 2018), robustly elevates BLA acetylcholine levels. Therefore, we hypothesized that these findings reflect a potentiation of NBM cholinergic projections to the BLA during withdrawal. In the present study, we use optogenetics and electrophysiology to understand how CIE exposure and withdrawal alter NBM cholinergic neuron excitability and measure the neurophysiological outcomes in the BLA. Our results indicate that CIE exposure and withdrawal enhances the excitability of NBM “cholinergic” neurons and increases NBM cholinergic neurotransmission in the BLA.

## Materials and Methods

### Animals

Adolescent male Sprague Dawley rats were purchased from Envigo and arrived at the Wake Forest University Medical School between postnatal day (P)35 and P40 (∼110–125 g). Lights in the animal facility were configured to a reverse 12/12 h light/dark cycle where lights automatically turned off between 9 A.M. and 9 P.M. Rodents were given *ad libitum* access to standard rat chow (LabDiet) and water. Animal welfare was monitored daily by laboratory staff and the Animal Resource Program (ARP). All experimental procedures were preapproved by the Institutional Animal Care and Use Committee (IACUC) at Wake Forest University School of Medicine.

### Stereotaxic viral microinjection surgeries

Rodents between postnatal days P38 and P43 were placed into an anesthesia induction chamber (Absolute Anesthesia) containing 2.0–2.5% isoflurane (Patterson Veterinary Supply) and 95% O_2_/5% CO_2_ (1.0 l/min) for 10 min. Animals were maintained under continuous 2.0–5.0% isoflurane exposure during the procedure. Bi-pedal reflexes were monitored before creating the incision and exposing the skull. The NBM was targeted using an automated stereotax system (Neurostar StereoDrive) with the following coordinates (in mm relative to bregma): NBM: anteroposterior (AP) −1.5 ± 0.5 mm; mediolateral (ML) 2.5 ± 0.5 mm; dorsoventral (DV) 7.2 ± 0.5 mm. Holes were drilled into the rodent skull with a Dremel, and injectors were slowly lowered into the appropriate position within neuronal tissue. We bilaterally injected channelrhodopsin (ChR2; rAAV5/hSyn-hChR2(H134R)-EYFP-WPRE; UNC Vector Core, University of North Carolina, Chapel Hill, NC), halorhodopsin (eNpHR; rAAV5/hSyn-eNpHR3.0-EYFP-WPRE; UNC Vector Core, University of North Carolina, Chapel Hill, NC), or ChrimsonR (red-shifted excitatory opsin; rAAV5/hSyn-ChrimsonR-tdTomato; Addgene #59171) into the NBM (0.5 μl/side) for 5 min (0.1 μl/min) using a syringe pump (Cole-Parmer). Injectors were left in place for an additional 5 min.

Rodents were given 2-ml warmed sterile saline (0.9% sodium chloride injection; Hospira) and an anti-inflammatory drug (1 mg/kg Meloxicam). After the incision was sutured and secured with skin adhesive (Skin Affix), rodents regained consciousness on a warm water blanket before returning to their home cage (single-housed). Laboratory staff monitored rodent health daily for one week following surgery until suture removal. Rodents were then pair-housed and recovered for four weeks to allow opsin expression at NBM terminals before experimentation. Injection sites were confirmed by collecting coronal slices of the NBM and visualizing EYFP (green) or tdTomato (red) using fluorescence microscopy postmortem. Rats with unintended viral placement were excluded from the study.

### Chronic intermittent ethanol vapor exposure

Pair-housed rodents remained in their home cages and were placed within Plexiglas vapor inhalation chambers (Triad Plastics) for the duration of the chronic intermittent ethanol (CIE) exposure paradigm. Ethanol was vaporized and continuously pumped into the inhalation chamber throughout the light cycle (12 h/d; 9 P.M. to 9:00 A.M.) for 10 consecutive days to produce repeated cycles of exposure and acute withdrawal. Rodents began the CIE chambers between postnatal days P66 and P71 and ended the CIE chambers between postnatal days P76 and P81. Laboratory personnel monitored animal health, and food pellets and water were discarded and replenished daily. Age-matched controls (denoted AIR in figures) were similarly housed but exposed to ambient air only. Blood samples were collected via tail snip twice throughout the exposure to measure blood ethanol concentrations (BECs) using a commercially available alcohol dehydrogenase/NADH (nicotinamide adenine dinucleotide plus hydrogen) enzymatic assay kit (Carolina Liquid Chemistries). The average BECs during the exposure was 229.3 ± 3.8 mg/dl (*n* = 102 rats). Rodents with average BECs outside the 150–275 mg/dl target range were excluded from the study. All whole-cell patch-clamp electrophysiology studies were completed following 24 h of withdrawal, between postnatal days P77 and P82.

### Brain slice preparation

Pairs of rats were anesthetized with isoflurane and were decapitated with a guillotine following the loss of their bi-pedal reflex. The brain was quickly removed and placed in ice-cold oxygenated sucrose artificial CSF (aCSF) solution that contained: 180 mm sucrose, 30 mm NaCl, 4.5 mm KCl, 1 mm MgCl_2_·6H_2_O, 26 mm NaHCO_3_, 1.2 mm NaH_2_PO_4_, 10 mm glucose, and 100 μm ketamine. Rodent coronal slices containing the NBM and BLA (400 μm thick) were prepared using a Leica VT1200/S vibrating microtome. Slices were incubated and equilibrated in an oxygenated standard aCSF solution at ∼28°C for 1 h that contained: 126 mm NaCl, 3 mm KCl, 1.25 mm NaH_2_PO_4_, 2 mm MgSO_4_·7H_2_O, 26 mm NaHCO_3_, 10 mm D-glucose, and 2 mm CaCl_2_·2H_2_O. All chemicals, including physostigmine (Tocris catalog #0622), mecamylamine (Tocris catalog #2843), and atropine (Sigma catalog #A0132), were purchased from Tocris Biosciences and Sigma-Aldrich.

### Whole-cell patch-clamp electrophysiology

Coronal slices were moved into a submersion-type recording chamber for whole-cell patch-clamp electrophysiology experiments that were continuously perfused with oxygenated, room temperature (∼25°C) standard aCSF at a rate of 2 ml/min using a peristaltic pump (MasterFlex). For NBM and BLA current-clamp recordings, recording electrodes were filled with a potassium gluconate intracellular solution that contained: 145 mm K-gluconate, 10 mm EGTA, 10 mm HEPES, 5 mm NaCl, 1 mm MgCl_2_·6H2O, 2 mm Mg-ATP, and 0.1 mm Na-GTP. The pH was adjusted to ∼7.2 − 7.3 with KOH. The osmolarity of this solution was ∼285 Osm/l. For all other recordings, glass electrodes were filled with a cesium gluconate intracellular solution that contained: 145 mm CsOH, 10 mm EGTA, 5 mm NaCl, 1 mm MgCl2·6H_2_O, 10 mm HEPES, 4 mm Mg-ATP, 0.4 mm Na-GTP, 0.4 mm QX314, and 1 mm CaCl_2_·2H_2_O. The pH was adjusted to ∼7.2–7.3 with D-gluconic acid, and the osmolarity was adjusted to ∼285 Osm/l. Data were acquired at 5 kHz and low-pass filtered at 2 kHz via an Axopatch 700B Amplifier and pClamp10.7 software (Molecular Devices).

Based on published studies, we used exclusion/inclusion criteria to distinguish NBM “cholinergic” neurons from “noncholinergic” neurons. “Cholinergic” neurons must exhibit the following characteristics: (1) slow and regular firing patterns; (2) afterhyperpolarization (AHP) duration following the first action potential must be ≥100 ms; and, (3) no rebound burst firing after a hyperpolarizing current step. This latter characteristic is a property of GABAergic neurons (Griffith and Matthews, 1986; Sotty et al., 2003). Although the NBM is an amorphous brain region, we targeted the NBM in coronal slices by placing the recording electrode just dorsal to the intersection between the lateral ventricle and ventrolateral striatum (NBM: AP −1.5 mm, ML ±2.5 mm, DV 7.2 mm). For these experiments, there were no pharmacological synaptic blockers added to the external aCSF. NBM neurons were injected with 600-ms current beginning at –100 pA and increasing by 25 pA every 20 s up to +300 pA. These recordings measured the AHP duration of the first action potential, the resting membrane potential (RMP), and the number of action potentials elicited from application of each current step. Additionally, we used a current ramp protocol beginning at –100 pA and increasing to +300 pA over 600 ms. The ramp protocol was repeated for five trials, where the first action potential was used to measure action potential properties like the action potential half-width and the peak amplitude (Price and McCool, 2022).

For BLA pyramidal neuron electrophysiology recordings, putative BLA principal/pyramidal neurons were distinguished from local GABAergic interneurons based on their characteristic low access resistance (<25 MΩ) and high membrane capacitance (>100 pF). We recorded neurons within the anterior/posterior subdivisions of the BLA as previous research indicates this region receives the densest NBM cholinergic input (Woolf and Butcher, 1982; Carlsen et al., 1985; Lee and Kim, 2019). Glutamatergic EPSCs were recorded from BLA neurons at a holding potential of −65 mV and were pharmacologically isolated with GABAA antagonist picrotoxin (100 μm) and APV (NMDA receptor antagonist, 50 μm). In separate recordings, GABAergic IPSCs were recorded at a holding potential of −10 mV and were pharmacologically isolated by adding glutamate receptor antagonists DNQX (AMPA/kainite receptor antagonist, 20 μm) and APV (50 μm) to the external aCSF. In addition, the nonselective mAChR antagonist atropine (Zwart and Vijverberg, 1997) was added to the external aCSF (500 nm) in a subset of recordings to isolate nAChR-dependent effects of NBM terminal activation/inhibition. Under the appropriate conditions, presynaptic nAChR-dependent modulation of glutamate release can serve as a proxy for circuit-level dysregulation of cholinergic NBM-BLA projections following chronic ethanol (Sizer et al., 2021).

Glutamatergic EPSCs were electrically evoked with platinum/iridium concentric bipolar stimulating electrodes (FHC Inc.) with an inner pole of 12.5 μm. The stimulating electrode was placed several hundred microns away from the basolateral nucleus within the stria terminalis (ST) fiber tract, just dorsal to the central amygdala and medial to the lateral/basolateral nuclei, to activate cortical and thalamic glutamatergic afferents. Modest electrical stimulation of the ST was normalized across all recordings to elicit synaptic currents with amplitudes of ∼100 pA to ensure these represent monosynaptic glutamatergic responses. Polysynaptic glutamatergic responses, likely because of an erroneous spread of electrical stimulation and subsequent activation of “local” circuits, were rarely encountered and excluded from data analysis. Direct activation of antidromic action currents within BLA principal neurons was never observed.

NBM terminals expressing channelrhodopsin (ChR2) were activated using a 473-nm, 100-mW DPSS blue laser (Bejing Viasho Technology Co, Ltd.). Halorhodopsin (NpHR; inhibitory opsin) and ChrimsonR (red-shifted excitatory opsin) were both activated using a 589-nm, 100-mW DPSS yellow laser (Bejing Viasho Technology Co, Ltd.). Lasers were connected to a fiber optic cable (Thorlabs); the naked end of the cable was placed just above the stria terminalis on the medial side of the BLA to activate/inhibit neurotransmitter release from NBM terminals. The 473/589-nm laser output was measured across a range of laser intensities with a photodiode (Switchable Gain Detector 320–1100 nm; Thorlabs) and an oscilloscope (Tektronix TDS1002B) to measure light intensity as voltage (V_out_ in mV). Laser intensity was expressed as input power (mW), calculated using the following formula: V_out_ = (R)(λ)(Transimpedence Gain)(Scale Factor)(Input Power)(1000), where R(λ) is defined as the responsivity of the photodiode.

To provide a measure of release probability, two electrical stimulations of equal intensity was delivered at an interstimulus interval of 50 ms. At short interstimulus intervals, the ratio between the first and second EPSC response amplitudes serves as a proxy for presynaptic neurotransmitter release probability (Andreasen and Hablitz, 1994; Dobrunz and Stevens, 1997). The paired-pulse ratio (PPR) was calculated using EPSC amplitudes as follows: [(Peak 2 amplitude)/(Peak 1 amplitude)]. For stria terminalis glutamate PPR recordings, the Peak 1 and Peak 2 amplitudes are distinct and were measured from the baseline preceding the first EPSC, as previously reported (Morales et al., 2018). In a subset of experiments (Fig. 3), we used optogenetics to activate/inhibit NBM terminal release with a yellow or blue laser (10 Hz, 5 ms) delivered 1 ms before electrical stimulation of stria terminalis inputs.

### Immunohistochemistry

Four weeks following stereotaxic microinjection of channelrhodopsin/eYFP into the NBM, rats (*n* = 16) underwent transcardial perfusions of PBS and 4% formalin for tissue fixation. Whole brains were extracted and stored in a 4% formalin solution at 4°C overnight. Brains were rinsed with a phosphate-buffered saline (0.12 m Na_2_HPO_4_, 0.18 m NaH_2_PO_4_, and 0.12 m NaCl) and placed in phosphate-buffered saline containing 30% sucrose solution for long-term storage at 4°C. This sucrose solution was replaced once a week to prevent bacterial growth. Coronal slices (50 μm thick) containing the NBM and the BLA were prepared using a Leica VT1200/S vibrating microtome. Unstained brain slices were immersed in 0.5 ml of cryoprotectant at −20°C for long-term storage. Fixed coronal slices were rinsed several times with 0.01 m PBS (Fisher Bioreagents) containing 0.3% Triton X-100 (PBS-Tx solution) and transferred into a blocking solution consisting of 5% normal donkey serum in PBS-Tx for 2 h. After rinsing with PBS-Tx, brain slices were incubated overnight with blocking solution (0.5 ml/well) containing chicken anti-GFP primary antibody (1:1000 dilution; Aves Labs; RRID: AB_10000240). Following three consecutive PBS-Tx rinses, brain slices were incubated in blocking solution containing Alexa Fluor 488 donkey anti-chicken secondary antibody (1:250 dilution; Jackson ImmunoResearch Labs; RRID: AB_2340375) for 1.5 h. Slices were repeatedly rinsed with PBS and mounted onto slides using ProLong Gold Antifade Mounting Media with DAPI (ThermoFisher). Coverslips were fixed to the slides and allowed to dry at 4°C overnight before imaging with a confocal microscope.

### Statistical analysis

Statistical analyses were completed using Prism 8 (GraphPad Software). Data were analyzed with repeated-measures two-way ANOVA, repeated-measures mixed-effects analysis, unpaired *t* tests, and Bonferroni’s *post hoc* tests depending on the experiment (see Table 1). A value of *p* < 0.05 was considered statistically significant, and statistical significance was denoted in the figures as follows: *p <* *0.05, **p <* *0.01, ***p <* *0.001, and ****p <* *0.0001. Graphs are represented as mean ± SEM.

**Table 1:** Results from statistical analyses

	Distribution (Shapiro–Wilk test)	Statistical test	95% CI
a	Max # of action potentials AIR: (W = 0.9109, *p* = 0.2501) CIE: (W = 0.8702, *p* = 0.0779)	Repeated-measures mixed effects analysis (AIR *n* = 11 cells) (CIE *n* = 11 cells)	[−9.179, 4.229]
b	AIR: (W = 0.8999, *p* = 0.1841) CIE: (W = 0.9601, *p* = 0.7728)	Unpaired *t* test (AIR *n* = 11 cells) (CIE *n* = 11 cells)	[0.95, 13.92]
c	AIR: (W = 0.9038, *p* = 0.2414) CIE: (W = 0.9219, *p* = 0.4082)	Unpaired *t* test (AIR *n* = 10 cells) (CIE *n* = 9 cells)	[−2.03, 21.82]
d	AIR: (W = 0.9507, *p* = 0.6765) CIE: (W = 0.9238, *p* = 0.4244)	Unpaired *t* test (AIR *n* = 10 cells) (CIE *n* = 9 cells)	[−0.66, 0.07]
e	Max # of action potentials AIR: (W = 0.8757, *p* = 0.0624) CIE: (W = 0.9375, *p* = 0.3874)	Repeated-measures mixed effects analysis (AIR *n* = 13 cells) (CIE *n* = 14 cells)	[−11.46, 5.557]
f	AIR: (W = 0.9477, *p* = 0.5636) CIE: (W = 0.9747, *p* = 0.9327)	Unpaired *t* test (AIR *n* = 13 cells) (CIE *n* = 14 cells)	[0.09, 13.92]
g	AIR: (W = 0.9762, *p* = 0.9635) CIE: (W = 0.9140, *p* = 0.2404)	Unpaired *t* test (AIR *n* = 12 cells) (CIE *n* = 12 cells)	[−10.23, 12.55]
h	AIR: (W = 0.9780, *p* = 0.9744) CIE: (W = 0.9127, *p* = 0.2309)	Unpaired *t* test (AIR *n* = 12 cells) (CIE *n* = 12 cells)	[−0.419, 0.538]
i	AIR: (W = 0.9214, *p* = 0.4575) CIE: (W = 0.8161, *p* = 0.1535)	Repeated-measures mixed effects ananlysis (AIR *n* = 20 cells) (CIE *n* = 11 cells)	[−0.09, 0.31]
j	AIR Physo: (W = 0.9475, *p* = 0.2814) CIE Physo: (W = 0.9516, *p* = 0.3920) AIR Opto: (W = 0.9906, *p* = 0.9988) CIE Opto: (W = 0.8977, *p* = 0.1730)	Two-way ANOVA Physostigmine (AIR *n* = 22 cells) (CIE *n* = 20 cells) Opto (AIR *n* = 20 cells) (CIE *n* = 11 cells)	[−25.95, −10.85]
k	AIR: (W = 0.9901, *p* = 0.9802) CIE: (W = 0.7989, *p* = 0.0793)	Repeated-measures mixed effects analysis (AIR *n* = 12 cells) (CIE *n* = 14 cells)	[−0.1439, 0.3015]
l	Baseline: (W = 0.9039, *p* = 0.4509) TTX: (W = 0.8826, *p* = 0.3498) 4-AP: (W = 0.9148, *p* = 0.5084)	Repeated-measures one-way ANOVA (AIR *n* = 4 cells)	Baseline vs TTX [789, 2301] Baseline vs 4-AP [−681.5, 830.2]
m	AIR: (W = 0.9564, *p* = 0.7315) CIE: (W = 0.9714, *p* = 0.9086)	Unpaired *t* test (AIR *n* = 12 cells) (CIE *n* = 8 cells)	[−0.5509, 0.7032]
n	AIR: (W = 0.9634, *p* = 0.8135) CIE: (W = 0.9061, *p* = 0.3274)	Repeated-measures mixed effects analysis (AIR *n* = 12 cells) (CIE *n* = 8 cells)	[4.821, 953.1]
o	Baseline: (W = 0.8142, *p* = 0.1053) TTX: (W = 0.9942, *p* = 0.9922) 4-AP: (W = 0.8697, *p* = 0.2652)	Repeated-measures one-way ANOVA (AIR *n* = 5 cells)	Baseline vs TTX [−63.72, −0.17] Baseline vs 4-AP [−36.29, 27.27]
p	AIR: (W = 0.9542, *p* = 0.3814) CIE: (W = 0.9417, *p* = 0.2578)	Unpaired *t* test (AIR *n* = 22 cells) (CIE *n* = 20 cells)	[−0.64, 1.16]
q	AIR: (W = 0.9664, *p* = 0.6481) CIE: (W = 0.9918, *p* = 0.8271)	Repeated-measures mixed effects ANOVA (AIR *n* = 6 cells) (CIE *n* = 5 cells)	[5.037, 44.60]
r	AIR: (W = 0.901, *p* = 0.3796) CIE: (W = 0.9130, *p* = 0.4566)	Repeated-measures mixed-effects analysis (AIR *n* = 22 cells) (CIE *n* = 20 cells)	[−0.07, 24.86]
s	AIR: (W = 0.8641, *p* = 0.9306) CIE: (W = 0.9306, *p* = 0.5559)	Unpaired *t* test (AIR *n* = 9 cells) (CIE *n* = 7 cells)	[−69.07, 37.75]
t	AIR (W = 0.9504, *p* = 0.5666) CIE: (W = 0.9391, *p* = 0.5433)	Repeated-measures two-way ANOVA (AIR *n* = 7) (CIE *n* = 5)	[−4.37, 0.35]
u	AIR (W = 0.9998, *p* = 0.9711) CIE: (W = 0.7962, *p* = 0.1056)	Repeated-measures two-way ANOVA (AIR *n* = 9) (CIE *n* = 6)	[−0.8789, 0.005]

## Results

### CIE and withdrawal enhance NBM “cholinergic” neuron excitability

Previous findings indicate that NBM cholinergic neurons can be distinguished from intermingled noncholinergic neurons based on their morphologic and electrophysiological properties. For example, cholinergic neurons have a large soma (Alonso et al., 1996; Williams et al., 1997; Bengtson and Osborne, 2000) and express low firing frequencies (Griffith and Matthews, 1986; Sim and Allen, 1998; Sotty et al., 2003; López-Hernández et al., 2017), which plateau at ∼10–20 Hz despite increasing current injections (Alonso et al., 1996; Khateb et al., 1992; Hedrick and Waters, 2010). Cholinergic neurons also contain a prolonged afterhyperpolarization (AHP) following individual action potentials (Griffith and Matthews, 1986; Unal et al., 2012). Although hyperpolarized cholinergic neurons have the capacity for burst firing (Khateb et al., 1992; Alonso et al., 1996), cholinergic neurons injected with a prolonged depolarizing current express slow and regular firing patterns (Khateb et al., 1992; Alonso et al., 1996; Sotty et al., 2003).

**Figure 1.**
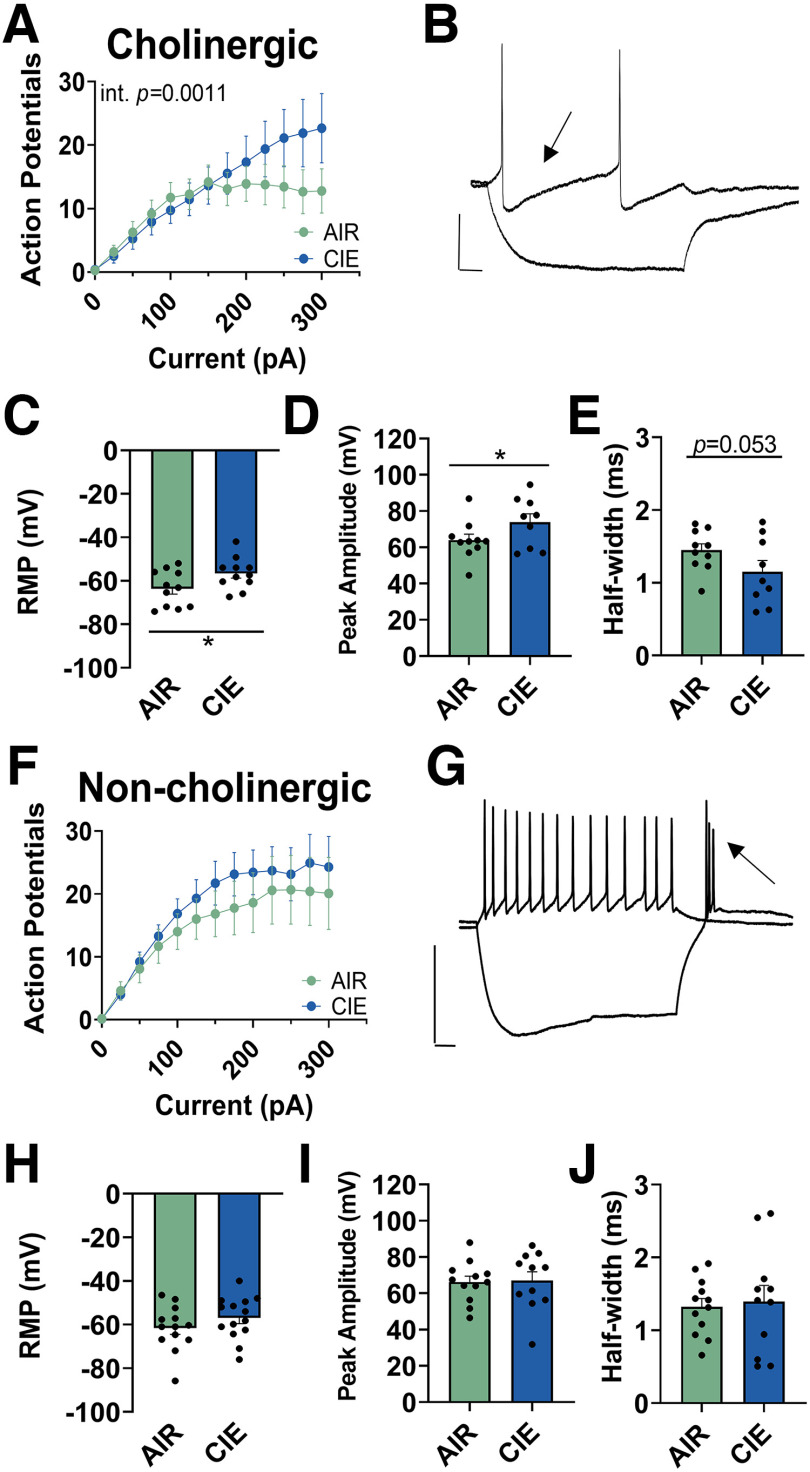
Withdrawal enhances the excitability of NBM “cholinergic” neurons. ***A***, CIE exposure and withdrawal increases the excitability of NBM “cholinergic” neurons (*n* = 11) compared with age-matched AIR controls (*n* = 11). Repeated-measures mixed-effects analysis, current X exposure interaction (*p *=* *0.0011). ***B***, Representative trace of 600-ms current steps of –100 and +25 pA in “cholinergic” neuron, with the arrow denoting the typical long afterhyperpolarization (AHP) duration. ***C***, Cholinergic neurons have increased resting membrane potential (AIR: *n* = 11, CIE: *n* = 11, unpaired *t* test, *p *=* *0.024), (***D***) increased peak amplitude (AIR: *n* = 10, CIE: *n* = 9, unpaired *t* test, *p *=* *0.049), and (***E***) a trend in decreased half-width (AIR: *n* = 10, CIE: *n* = 9, unpaired *t* test, *p *=* *0.053) during withdrawal (***F***) CIE exposure and withdrawal does not alter the excitability of NBM “noncholinergic” neurons (*n* = 14) compared with AIR controls (*n* = 13). Repeated-measures mixed-effects analysis (no current X CIE interaction, *p *=* *0.94). ***G***, Representative trace of 600-ms current steps of –100 and +25 pA in “noncholinergic” neuron, with the arrow denoting burst firing after hyperpolarizing current ends. Scale bars: *y*-axis 20 mV and *x*-axis 50 ms for all traces in figure. ***H***, Noncholinergic neurons show no changes in resting membrane potential (AIR: *n* = 13, CIE: *n* = 14, unpaired *t* test, *p *=* *0.119), (***I***) peak amplitude (AIR: *n* = 12, CIE: *n* = 11, unpaired *t* test, *p *=* *0.417), or (***J***) half-width (AIR: *n* = 12, CIE: *n* = 11, unpaired *t* test, *p *=* *0.400) during withdrawal. *p < 0.05. *Figure Contributions*: Sarah E. Sizer, Michaela E. Price, and Brian C. Parrish performed the experiments. Sarah E. Sizer analyzed the data.

Based on these published findings, we used three criteria to distinguish putative “cholinergic” neurons from “noncholinergic” (GABAergic and glutamatergic) neurons in the NBM (see Materials and Methods). We performed current-clamp electrophysiology recordings from NBM neurons in AIR-exposed and CIE-exposed rodents to understand whether chronic ethanol exposure and withdrawal alter NBM “cholinergic” neuron properties. We found that NBM neurons classified as “cholinergic” show enhanced excitability during withdrawal (*n* = 11 cells from 6 pairs of rats) relative to age-matched AIR controls (*n* = 11 cells from 8 pairs of rats). Repeated-measures mixed-effects analysis (main effect of current *F*_(12,225)_ = 21.50, *p *<* *0.0001; current X CIE interaction *F*_(12,225)_ = 2.855, *p *=* *0.0011; Fig. 1*A*^a^). CIE exposure and withdrawal also depolarized the resting membrane potential (AIR: −63.6 ± 8.3 mV, *n* = 11 cells from 11 pairs of rats; CIE: −56.7 ± 7.3 mV, *n* = 11 cells from 8 pairs of rats; unpaired *t* test, *t*_(20)_ = 2.115, *p *=* *0.024; Fig. 1*C*^b^), increased the action potential peak amplitude (AIR: 63.9 ± 10.7 mV, *n* = 10 cells; CIE: 73.8 ± 13.9 mV, *n* = 9 cells; unpaired *t* test, *t*_(17)_ = 1.751, *p *=* *0.049; Fig. 1*D*^c^), and reduced the action potential half-width (AIR: 1.4 ± 0.3 ms, *n* = 10 cells; CIE: 1.1 ± 0.5 ms, *n* = 9 cells; unpaired *t* test, *t*_(17)_ = 1.706, *p *=* *0.053; Fig. 1*E*^d^) of NBM “cholinergic neurons.” In contrast, “noncholinergic” neurons exhibited no significant differences in any of these measures (AIR: *n* = 13 cells; CIE: *n* = 14 cells; repeated-measures mixed-effects analysis; main effect of current *F*_(12,332)_ = 26.37, *p *<* *0.0001; no current X CIE interaction *F*_(12,332)_ = 0.451, *p *=* *0.94; Fig. 1*F*^e^), as well as resting membrane potential (AIR: −61.6 ± 10.4 mV, *n* = 13 cells; CIE: −56.9 ± 9.7 mV, *n* = 14 cells; unpaired *t* test, *t*_(25)_ = 1.209, *p *=* *0.119; Fig. 1*H*^f^), action potential peak amplitude (AIR: 66.3 ± 11.2 mV, *n* = 12 cells; CIE: 67.4 ± 15.4 mV, *n* = 12 cells; unpaired *t* test, *t*_(22)_ = 0.212, *p *=* *0.417; Fig. 1*I*^g^), and half-width (AIR: 1.3 ± 0.4 ms, *n* = 12 cells; CIE: 1.4 ± 0.7 ms; *n* = 12 cells; unpaired *t* test, *t*_(22)_ = 0.2556, *p *=* *0.400; Fig. 1*J*^h^). These data suggest that withdrawal increases NBM “cholinergic” neuron neuronal excitability. Since it is well-established that NBM “cholinergic” neurons project to the BLA (AE Power and McGaugh, 2002; Jiang et al., 2016; Aitta-Aho et al., 2018; Crouse et al., 2020), we hypothesized that enhanced NBM “cholinergic” neuron excitability during ethanol withdrawal might have downstream effects on cholinergic modulation of local BLA circuits.

### Tonic activation of presynaptic nAChRs facilitates glutamate release at stria terminalis inputs during ethanol withdrawal

**Figure 2.**
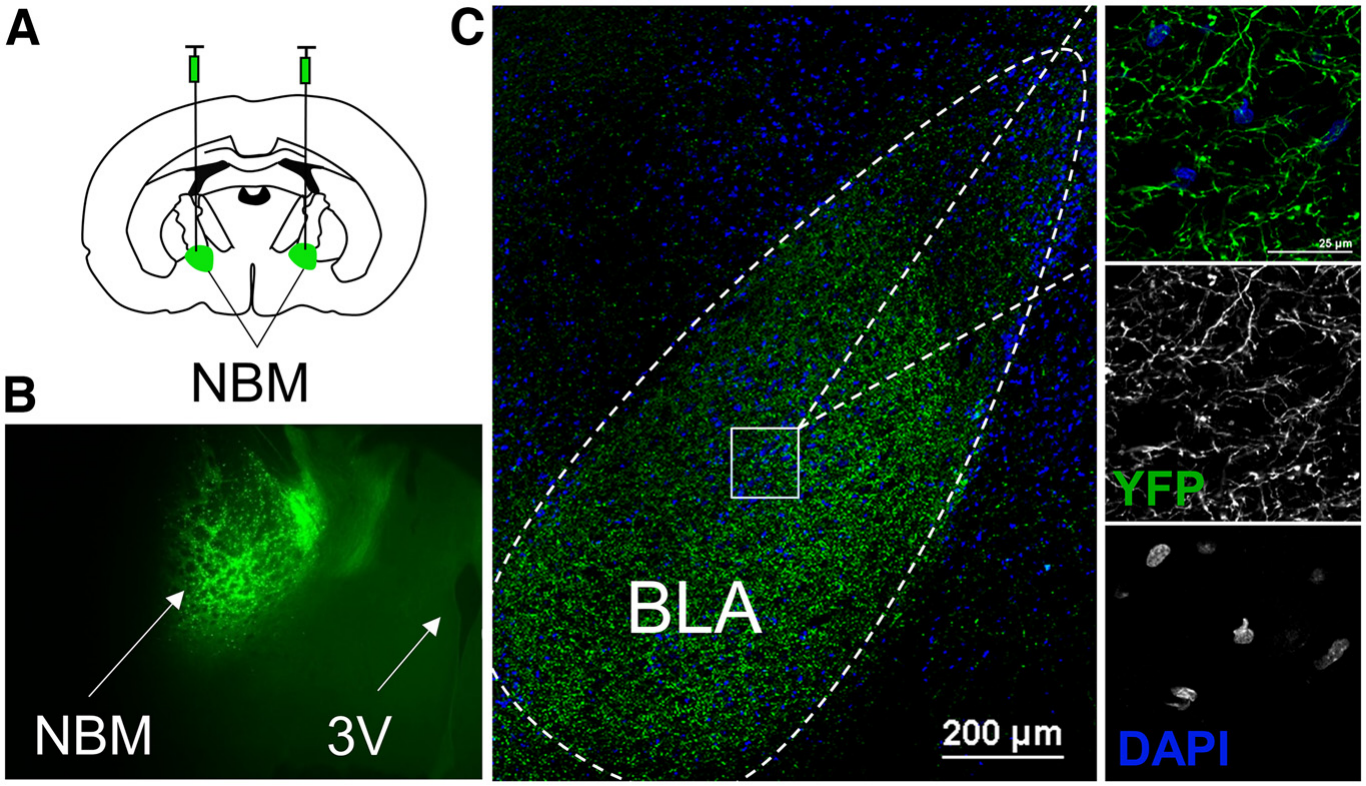
Immunohistochemical validation of opsin expression in NBM-BLA projection neurons. ***A***, Schematic illustrating microinjection of AAV5-hSyn-ChR2(H134R)-eYFP into the NBM (AP −1.5 mm, ML ±2.5 mm, DV 7.2 mm). ***B***, Representative fluorescent 4× image of NBM injection site and (***C***) representative fluorescent 20× image (left) of YFP^+^ NBM terminal fields in the BLA following a four-week recovery period. 60× images (right) illustrate opsin expression within NBM afferents (YFP) and not the soma of BLA neurons (DAPI). BLA, basolateral amygdala; NBM, nucleus basalis magnocellularis; 3V, third ventricle. *Figure Contributions*: Sarah E. Sizer, Kimberly F. Raab-Graham, Chelcie F. Heaney, and Samuel H. Barth performed the experiments.

Therefore, we used optogenetics to manipulate NBM terminal activity in AIR-exposed and CIE-exposed rodents and measure the neurophysiological outcomes at distinct neuronal populations in the BLA. We microinjected adeno-associated viral vectors containing channelrhodopsin (AAV5-hSyn-ChR2(H134)-EYFP), ChrimsonR (red-shifted excitatory opsin; AAV5-hSyn-ChrimsonR-tdTomato), or halorhodopsin (AAV5-hSyn-eNpHR3.0-EYFP) into the NBM (AP −1.5 mm, ML ±2.5 mm, DV 7.2 mm; AE Power and McGaugh, 2002; AE Power et al., 2002, 2003). Fluorescence images show robust YFP fluorescence at the injection site (Fig. 2*B*) and NBM terminal fields (Fig. 2*C*) concentrated in the basolateral nucleus four weeks after channelrhodopsin injection. YFP expression is consistent with previous reports of acetylcholinesterase staining in the BLA (Woolf and Butcher, 1982).

**Figure 3.**
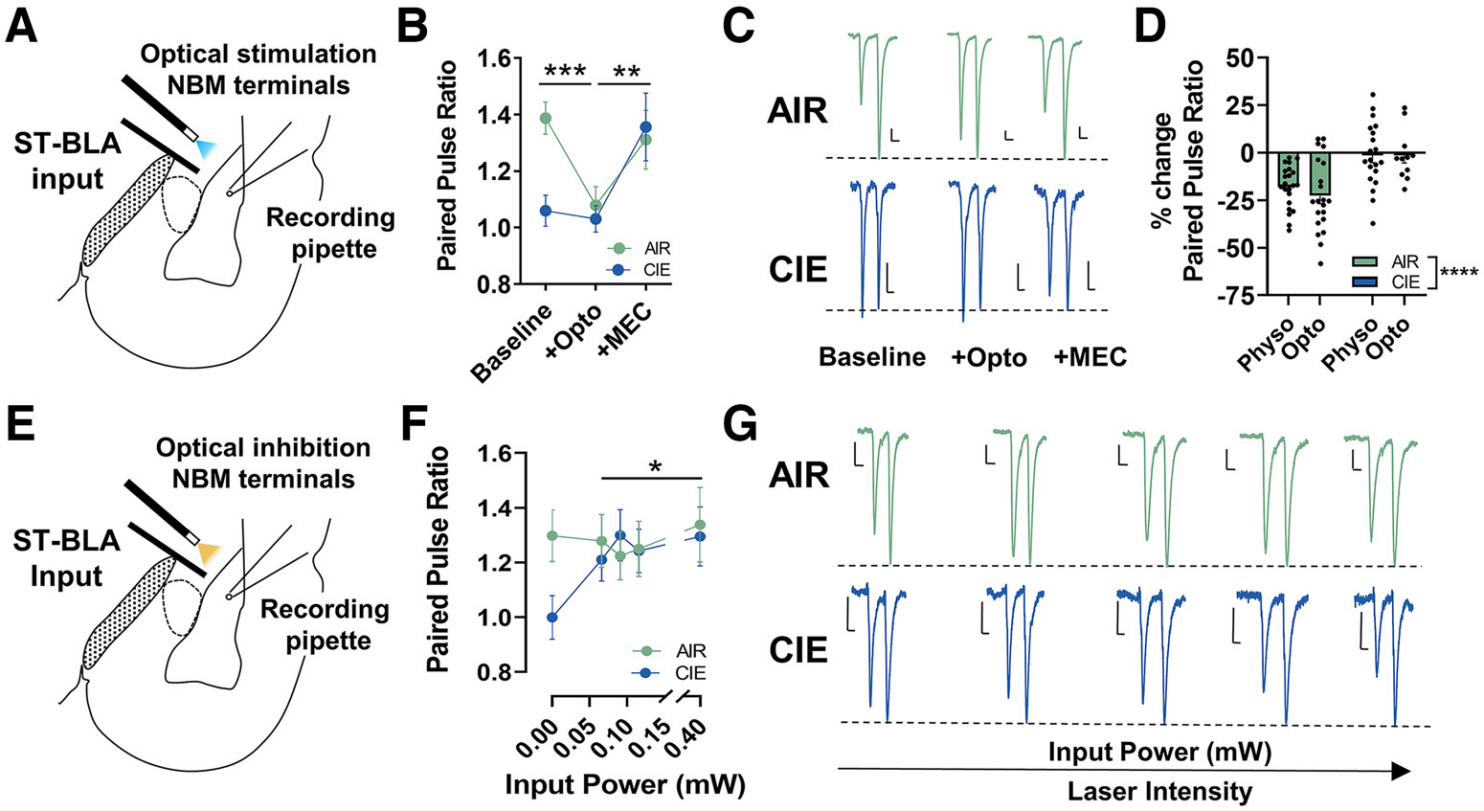
Tonic activation of presynaptic nicotinic acetylcholine receptors facilitates pathologic glutamate release at stria terminalis inputs during withdrawal. ***A***, Schematic illustrating placement of electrical stimulation of stria terminalis (ST) afferents and optical stimulation of NBM terminals while recording from BLA pyramidal neurons. ***B***, Optical stimulation (10 Hz, 5 ms) of NBM terminals with 473-nm laser before electrical stimulation of ST afferents increases glutamate release (decreases paired-pulse ratio) in AIR neurons (*n* = 20), with no effect in CIE neurons (*n* = 11). Mecamylamine (MEC; nonselective nAChR antagonist; 100 μm) perfusion illustrates presynaptic nicotinic receptors mediate this effect [repeated-measures two-way ANOVA, main effect of laser stimulation + MEC (*p *=* *0.0002) and laser X exposure interaction (*p *=* *0.012)]. Bonferroni’s *post hoc* tests show significant differences in AIR: baseline versus opto (***p =* *0.0004), opto versus MEC (**p =* *0.0091), and CIE: opto versus MEC (**p =* *0.0091) and baseline versus MEC (*p *=* *0.015). ***C***, Representative traces of paired-pulse ratios (PPRs) in AIR (green) and CIE (purple) neurons. ***D***, Upregulation of synaptic acetylcholine levels with the acetylcholinesterase antagonist physostigmine (0.5 μm) causes similar effects on the PPR as optical stimulation of NBM terminals in AIR and CIE animals (Opto AIR: *n* = 20 cells, Opto CIE: *n* = 11 cells, physostigmine AIR: *n* = 23 cells, physostigmine CIE: *n* = 21 cells). Two-way ANOVA; significant main effect of CIE (****p <* *0.0001), no significant effect of method of acetylcholine upregulation (*p *=* *0.3202), no significant CIE X acetylcholine interaction (*p *=* *0.7769). ***E***, Schematic illustrating placement of electrical stimulation of ST afferents and optical inhibition of NBM terminals. ***F***, 589-nm laser inhibition (10 Hz, 5 ms) of NBM terminals at increasing laser intensity (measured as input power, mW) reverses pathologic glutamate release at ST afferents in CIE neurons (*n* = 14), with no effect in AIR neurons (*n* = 12). Mixed-effects analysis, laser X exposure interaction. Bonferroni’s *post hoc* tests reveal a significant increase in PPR in CIE neurons following laser inhibition of NBM terminals relative to baseline (*p <* *0.05 see Results for stats). ***G***, Representative traces of PPRs in AIR and CIE neurons with increasing laser intensity. Scale bars: *y*-axis 20 pA and *x*-axis 20 ms for all traces in figure. *Figure Contributions*: Sarah E. Sizer performed the experiments. Sarah E. Sizer analyzed the data.

Several studies have shown that CIE exposure and withdrawal increase the glutamate release probability (decrease the paired-pulse ratio) of electrically-evoked stria terminalis afferents (Christian et al., 2013; Morales et al., 2018). In fact, this effect may be due in part to tonic activation of presynaptic α_7_ nicotinic acetylcholine receptors during withdrawal (Sizer et al., 2021). Therefore, we hypothesized that upregulation of NBM terminal activity helps produce pathologic glutamate release by activating presynaptic nicotinic acetylcholine receptors (nAChRs) expressed by stria terminalis synapses. We used optogenetics to activate channelrhodopsin or chrimson-expressing NBM terminals before electrical stimulation of stria terminalis paired-pulse ratios (PPR) to understand the effect of NBM terminal activity on glutamate release probability. The recordings concluded with a mecamylamine drug wash (MEC; nonselective nAChR antagonist; 100 μm) to determine whether NBM terminal stimulation activated presynaptic nicotinic receptors at stria terminalis synapses. The effect of NBM terminal activation and effects of MEC perfusion was the same regardless of gating by Channelrhodopsin or ChrimsonR. We therefore collapsed the AIR and CIE data across these excitatory opsins for Figure 3*B*^i^ (channelrhodopsin: *n* = 14 cells; chrimson *n* = 6 cells; repeated-measures two-way ANOVA; main effect of opto + MEC *F*_(2,36)_ = 6.806, *p *=* *0.003; no main effect of opsin *F*_(1,18)_ = 2.765, *p *=* *0.114; no opto + MEC X opsin interaction *F*_(2,36)_ = 0.5656, *p *=* *0.573). As previously reported, CIE exposure and withdrawal significantly increase stria terminalis glutamate release probability (decrease PPR) at baseline (AIR: 1.4 ± 0.1, *n* = 20 cells from 9 pairs of rats; CIE: 1.1 ± 0.1, *n* = 11 cells from 5 pairs of rats, planned unpaired *t* test, *t*_(29)_ = 3.735, *p *=* *0.004; Fig. 3*B*^i^). Laser stimulation of NBM terminals increases the stria terminalis glutamate release probability in AIR neurons, with no effect in CIE neurons (repeated-measures two-way ANOVA; main effect of opto + MEC, *F*_(2,28)_ = 9.935, *p *= 0.0002; significant opto + MEC X CIE interaction *F*_(2,28)_ = 4.738, *p *=* *0.012; Fig. 3*B*^i^). Bonferroni’s multiple comparison test illustrates significant differences in AIR baseline versus opto (*t *=* *4.112, *p *=* *0.0004), but no significant differences in CIE baseline versus opto (*t *=* *0.2855, *p *>* *0.999). Pharmacological upregulation of synaptic acetylcholine levels with physostigmine (0.5 μm; acetylcholinesterase antagonist) cause similar alterations in stria terminalis release probability (opto AIR: *n* = 20 cells from 9 pairs of rats, opto CIE: *n* = 11 cells from 5 pairs of rats, physostigmine AIR: *n* = 23 cells from 12 pairs of rats, physostigmine CIE: *n* = 21 cells from 9 pairs of rats; two-way ANOVA; significant main effect of CIE *F*_(1,71)_ = 19.74, *p *<* *0.0001; no significant effect of method of acetylcholine upregulation *F*_(1,71)_ = 1.002, *p *=* *0.3202; no significant CIE X acetylcholine *F*_(1,71)_ = 0.0809, *p *=* *0.7769; Fig. 3*D*^j^). MEC perfusion reversed the effect of laser stimulation in AIR neurons to baseline levels (opto vs MEC, *t *=* *3.096, *p *=* *0.0091; baseline vs MEC, *t *=* *1.016, *p *=* *0.941), while MEC reverses pathologic glutamate release in CIE neurons to AIR control levels (opto vs MEC, *t *=* *3.213, *p *=* *0.0064; baseline vs MEC, *t *=* *2.928, *p *=* *0.015). These data suggest that increases in NBM terminal release upregulate presynaptic nAChR activity and facilitate pathologic glutamate release at stria terminalis inputs during withdrawal.

Based on these findings, we hypothesized that laser inhibition of halorhodopsin-expressing NBM terminals before electrical stimulation of stria terminalis afferents would reverse elevated glutamate release during withdrawal. Planned unpaired *t* test show significant differences in AIR and CIE paired-pulse ratios at baseline (AIR: 1.3 ± 0.1, *n* = 12 cells from 6 pairs of rats; CIE: 1.0 ± 0.1, *n* = 14 cells from 6 pairs of rats; planned unpaired *t* test, *t*_(24)_ = 2.429, *p *=* *0.012; Fig. 3*F*^k^). Inhibition of NBM terminal activity with increasing 589-nm laser intensity (expressed as input power, mW) significantly increases the PPR in CIE neurons, with no effect in AIR neurons (repeated-measures mixed-effects analysis; laser X CIE interaction *F*_(4,78)_ = 2.687, *p *=* *0.037 Fig. 3*F*^k^). Bonferroni’s multiple comparisons *post hoc* test show increases in CIE PPR (CIE: baseline vs 0.07-mW input power, *t *=* *2.838, *p *= 0.023; baseline vs 0.09-mW input power, *t *=* *3.462, *p *=* *0.004; baseline vs 0.12mW input power, *t *=* *2.748, *p *=* *0.030; baseline vs 0.39-mW input power, *t *=* *3.145, *p *=* *0.009), with no effect in AIR PPR (AIR: baseline vs 0.07-mW input power, *t *=* *0.2398, *p *>* *0.999; baseline vs 0.09-mW input power, *t *=* *0.9510, *p* > 0.999; baseline vs 0.12mW input power, *t *= 0.6541, *p *>* *0.999; baseline vs 0.39-mW input power, *t *=* *0.4994, *p *>* *0.999). Collectively, these findings show that inhibiting NBM terminal activity restores glutamate release at stria terminalis afferents during withdrawal to levels that are similar to AIR controls.

### Ethanol withdrawal differentially alters NBM cholinergic/glutamatergic, and NBM GABAergic neurotransmission onto BLA pyramidal neurons

**Figure 4.**
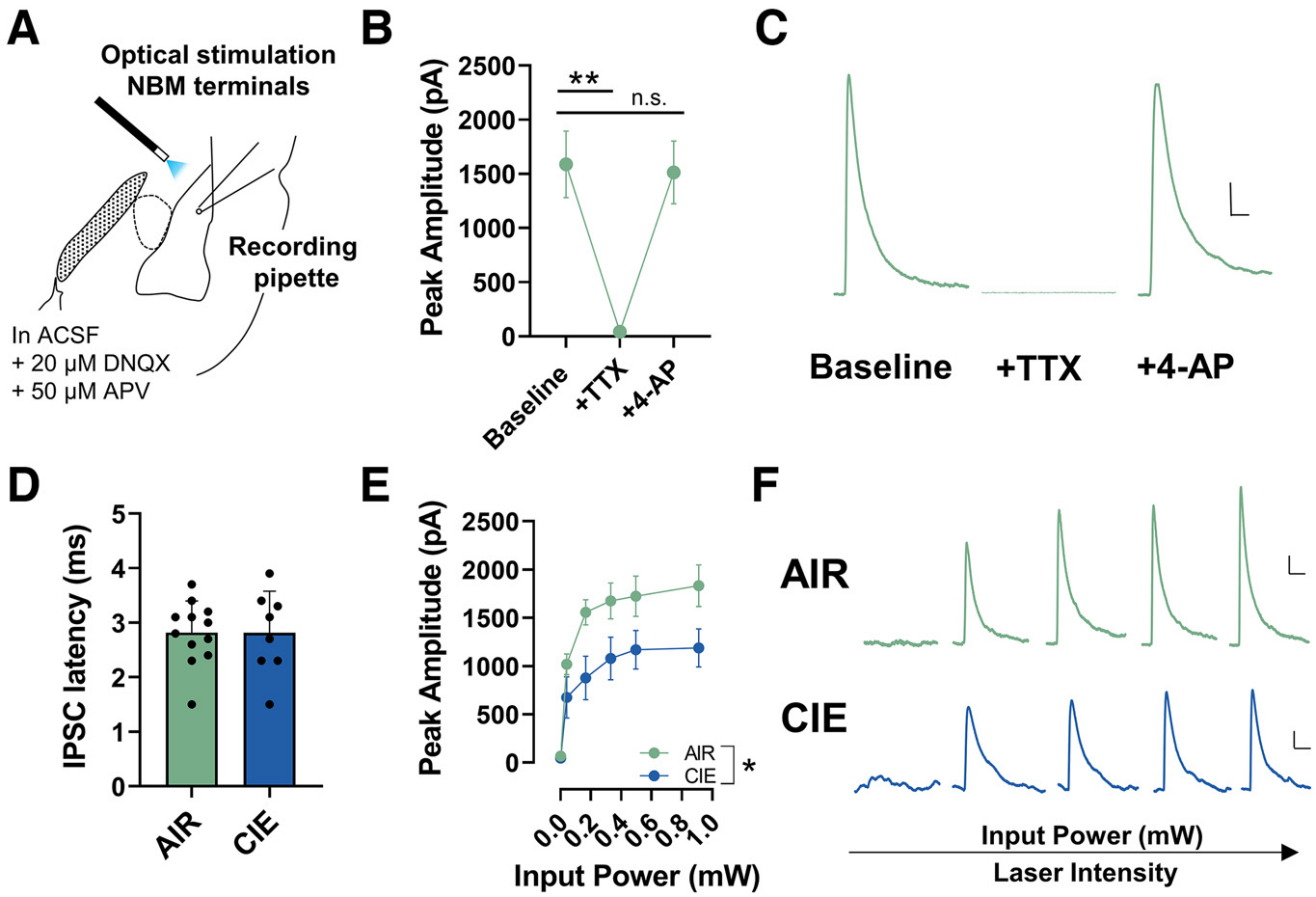
CIE exposure and withdrawal decrease IPSCs elicited from NBM GABAergic terminals. ***A***, Schematic illustrating placement of 473-nm laser for optical activation of NBM GABAergic neuron terminals. ***B***, TTX (1 μm) perfusion occludes IPSCs and is fully recovered with 4-AP (1 mm) perfusion in AIR neurons (*n* = 4 cells; repeated-measures one-way ANOVA; *p *=* *0.002), Bonferroni’s multiple comparisons test (baseline vs TTX, **p =* *0.002 and baseline vs 4-AP, *p *>* *0.999). ***C***, Representative traces of optically evoked IPSCs in AIR neurons following TTX and 4-AP perfusion. ***D***, No significant differences in IPSC latency between AIR (*n* = 12) and CIE (*n* = 8) neurons (unpaired *t* test, *p* = 0.400). ***E***, Activation of GABAergic terminals with 473-nm laser (5 ms) elicits a large GABAergic IPSC in AIR (*n* = 12) and CIE (*n* = 8) neurons. Mixed effects analysis showed main effect of laser stimulation (*p *<* *0.001), main effect of exposure (*p =* *0.048), and laser X exposure interaction (*p *=* *0.031). ***F***, Representative traces of AIR neurons and CIE neurons. Scale bars: *y*-axis 200 pA and *x*-axis 50 ms for all traces in figure. *Figure Contributions*: Sarah E. Sizer performed the experiments. Sarah E. Sizer analyzed the data.

Previous work estimates that 10% of NBM projections to the BLA are GABAergic (Mascagni and McDonald, 2009). However, no electrophysiology studies have measured the physiological significance of NBM GABAergic projections onto BLA pyramidal neurons. Therefore, we measured optically-evoked IPSCs elicited from channelrhodopsin-expressing NBM terminals. Optical stimulation of NBM terminals produced robust GABAergic IPSCs. Perfusion of the sodium channel blocker tetrodotoxin (TTX; 1 μm) abolished the NBM IPSCs and subsequent application of potassium channel blocker 4-AP (20 mm) reversed this effect (AIR: *n* = 4 cells from 3 pairs of rats; repeated-measures one-way ANOVA, *F*_(2,6)_ = 23.42, *p *= 0.002; Fig. 4*B*^m^). Bonferroni’s multiple comparison test shows significant differences in baseline versus TTX (*t *=* *6.068, *p *=* *0.002) and no significant difference in baseline versus 4-AP (*t *=* *0.2920, *p *>* *0.999). Additionally, optically-evoked IPSCs were characterized by short latencies that were not significantly different between AIR and CIE neurons (AIR: 2.8 ± 0.2, *n* = 12 cells from 6 pairs of rats; CIE: 2.8 ± 0.3, *n* = 8 cells from four pairs of rats; unpaired *t* test, *t*_(18)_ = 0.0139, *p *=* *0.495; Fig. 4*D*^n^). These data all suggest that the NBM-mediated IPSCs were monosynaptic. Finally, we measured the IPSC amplitude across a range of laser intensities (expressed as input power, mW) and found that CIE exposure significantly attenuated the IPSC amplitudes relative to AIR controls (AIR: *n* = 12 cells from 6 pairs of rats; CIE: *n* = 8 cells from four pairs of rats; repeated-measures mixed-effects analysis; main effect of laser intensity *F*_(5,89)_ = 52.41, *p *<* *0.001; main effect of CIE *F*_(1,18)_ = 4.504, *p *=* *0.048; laser X CIE interaction *F*_(5,89)_ = 2.600, *p *=* *0.031; Fig. 4*E*^o^). There were no significant differences in access resistance between AIR and CIE neurons (data not shown; AIR: 9.3 ± 0.7 MΩ, *n* = 12 cells from 6 pairs of rats; CIE: 10.3 ± 1.8 MΩ, *n* = 8 cells from four pairs of rats; unpaired *t* test, *t*_(18)_ = 0.5872, *p *=* *0.282).

**Figure 5.**
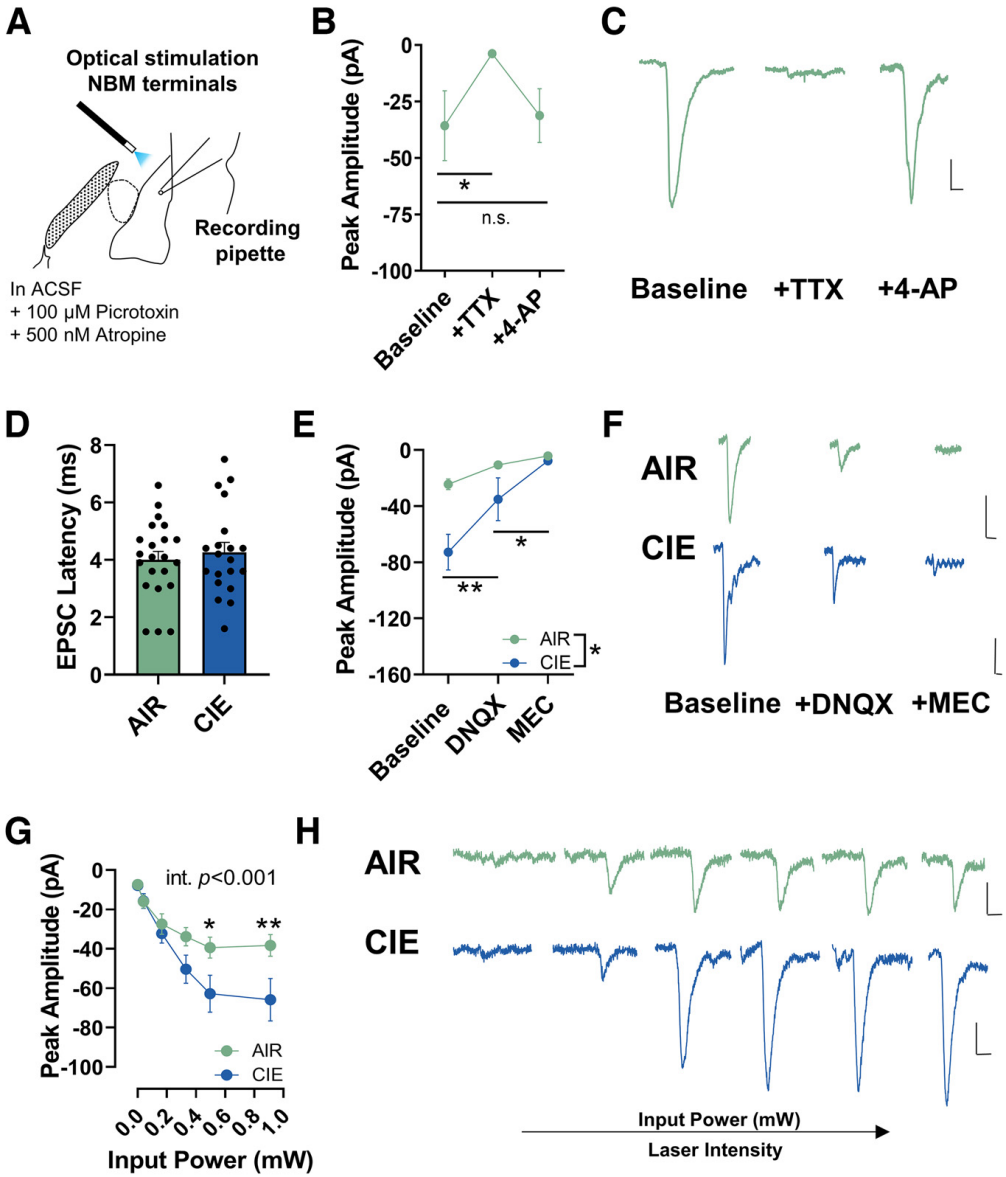
CIE exposure and withdrawal potentiates monosynaptic glutamatergic and nicotinic EPSCs elicited from NBM terminals. ***A***, Schematic illustrating placement of 473-nm laser for optical activation of NBM terminals while recording from BLA pyramidal neurons. ***B***, TTX (1 μm) perfusion occludes EPSC and is fully recovered with 4-AP (1 mm) perfusion in AIR neurons (*n* = 5 cells; repeated-measures one-way ANOVA; *p *=* *0.050), Bonferroni’s multiple comparisons test (baseline vs TTX, *p = 0.049 and baseline vs 4-AP, *p *=* *0.99). ***C***, Representative traces of NBM EPSCs following TTX and 4-AP perfusion. ***D***, No significant differences in EPSC latency in AIR (*n* = 20) and CIE (*n* = 22) neurons (unpaired *t* test, *p *=* *0.282) EPSC latency and TTX + 4-AP data indicate that the optically evoked NBM EPSCs are monosynaptic. ***E***, DNQX (AMPA/kainate receptor antagonist; 20 μm) and mecamylamine (MEC; nonselective nicotinic receptor antagonist; 100 μm) ablate optically evoked EPSC in AIR (*n* = 7) and CIE neurons (*n* = 5). Mixed-effects analysis, main effect of CIE exposure (*p =* *0.016), main effect of DNQX + MEC (*p *=* *0.0002), CIE X DNQX + MEC interaction (*p *=* *0.0303). Bonferroni’s multiple comparisons test (CIE baseline vs DNQX, **p =* *0.0047 and DNQX vs MEC, *p =* *0.0395). ***F***, Representative traces of AIR and CIE EPSCs following DNQX and MEC perfusion. ***G***, Optical excitation of NBM terminals at increasing laser intensities [measured as input power (mW)] in AIR (*n* = 22) and CIE (*n* = 20) neurons. Mixed-effects analysis showed main effect of laser (*p *<* *0.0001), trending main effect of exposure (*p *=* *0.051), and laser X exposure interaction (*p *=* *0.0005). Bonferroni’s *post hoc* tests show significant differences in AIR and CIE EPSCs at the two highest laser intensities (*p =* *0.023 and **p <* *0.002). ***H***, Representative traces of EPSCs in AIR and CIE neurons. Scale bars: *y*-axis 20 pA and *x*-axis 20 ms for all traces in figure. *Figure Contributions*: Sarah E. Sizer performed the experiments. Sarah E. Sizer analyzed the data.

NBM cholinergic terminals also directly synapse onto BLA pyramidal neurons and may co-release glutamate. Therefore, we measured optically-evoked EPSCs from NBM terminals and found that optical stimulation of NBM terminals produced a modest, EPSC. Again, perfusion of the sodium channel blocker tetrodotoxin (TTX; 1 μm) abolished the NBM EPSCs while application of potassium channel blocker 4-AP (20 mm) reversed this effect (AIR: *n* = 5 cells from 3 pairs of rats; repeated-measures one-way ANOVA, *F*_(2,8)_ = 4.481, *p *=* *0.050; Fig. 5*B*^o^). Bonferroni’s multiple comparison test shows significant differences in baseline versus TTX (*t *=* *2.766, *p *=* *0.049) and no significant difference in baseline versus 4-AP (*t *=* *0.3908, *p *=* *0.99). We measured the EPSC latency and found no significant differences between AIR and CIE neurons (AIR: 4.0 ± 0.3, *n* = 22 cells from 7 pairs of rats; CIE: 4.3 ± 0.3, *n* = 20 cells from 7 pairs of rats; unpaired *t* test, *t*_(40)_ = 0.5823, *p *=* *0.282; Fig. 5*D*^p^). Together, the EPSC latency and TTX + 4-AP data strongly indicate that the optically-evoked NBM EPSCs are monosynaptic. In a separate experiment, DNQX (AMPA/kainite receptor antagonist; 20 μm) and mecamylamine (MEC; nonselective nicotinic acetylcholine receptor antagonist; 100 μm) perfusion ablated the EPSCs released from NBM terminals in AIR and CIE neurons [AIR: *n* = 7 cells from 6 pairs of rats; CIE: *n* = 5 cells from 3 pairs of rats; mixed-effects analysis shows main effect of CIE exposure (*F*_(1,10)_ = 8.346, *p *=* *0.016), main effect of DNQX + MEC (*F*_(2,15)_ = 15.27, *p *=* *0.0002), and CIE X DNQX + MEC interaction (*F*_(2,15)_ = 4.456, *p *=* *0.0303); Fig. 5*E*^q^]. Bonferroni’s *post hoc* test show significant differences in CIE baseline versus DNQX (*t *=* *3.850, *p *=* *0.0047) and CIE DNQX versus MEC (*t *=* *2.804, *p *=* *0.0395). These findings indicate that optically-evoked NBM EPSCs contain both nicotinic and glutamatergic components. Although published work suggests that ∼35% of ChAT^+^ NBM-BLA projections may co-release acetylcholine and glutamate (Allen et al., 2006; Nickerson Poulin et al., 2006), it remains unclear whether the glutamatergic EPSCs originate from small populations of NBM glutamatergic projections or cholinergic neurons that co-release glutamate.

We recorded from BLA pyramidal neurons and measured optically-evoked EPSCs from channelrhodopsin-expressing NBM terminals to understand whether CIE exposure and withdrawal upregulates NBM terminal activity in the BLA. We measured the EPSC amplitude across a range of laser intensities (expressed as input power, mW) and found that CIE exposure significantly increases the EPSC amplitude relative to AIR controls (AIR: *n* = 22 cells from 7 pairs of rats; CIE: *n* = 20 cells from 7 pairs of rats; repeated-measures mixed-effects analysis; main effect of laser intensity *F*_(5,192)_ = 41.91, *p *<* *0.0001; trending main effect of CIE *F*_(1,40)_ = 4.041, *p *=* *0.051; laser X CIE interaction *F*_(5,192)_ = 4.674, *p *=* *0.0005; Fig. 5*G*^r^). Bonferroni’s *post hoc* tests show significant differences between AIR and CIE EPSCs at the two highest laser intensities (input power: 0.50 mW, *t *=* *2.921, *p *=* *0.023; Input power: 0.91mW; *t *=* *3.588, *p *=* *0.002). Withdrawal-induced increases in EPSC amplitude were not because of significant differences in access resistance between AIR and CIE neurons (data not shown; AIR: 11.1 ± 0.6MΩ, *n* = 22 cells from *n* = 7 pairs of rats; CIE: 12.6 ± 1.1MΩ, *n* = 20 cells from 7 pairs of rats; unpaired *t* test, *t*_(18)_ = 1.18, *p *=* *0.123).

### Upregulation of glutamatergic and cholinergic NBM terminals increases BLA pyramidal neuron firing during withdrawal

**Figure 6.**
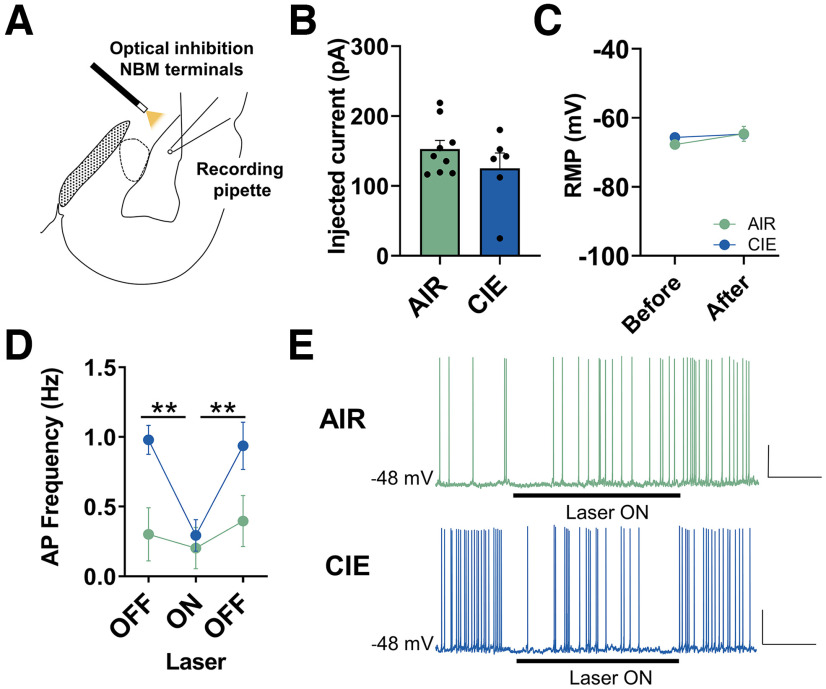
NBM terminal activity upregulates BLA pyramidal neuron firing following CIE exposure and withdrawal. ***A***, Schematic illustrating placement of 589-nm laser for optical inhibition of NBM terminals expressing halorhodopsin while recording from BLA pyramidal neurons. BLA pyramidal neurons were injected with depolarizing current to a membrane potential of –48 mV. Action potential firing frequency was recorded for 30-s baseline (laser OFF), during 60-s laser inhibition of NBM terminals (laser ON), and 30-s recovery (laser ON). ***B***, Depolarizing current injected to reach –48-mV membrane potential is not significantly different in AIR (*n* = 9 cells) and CIE neurons (*n* = 6 cells), unpaired *t* test (*p *=* *0.131). ***C***, Resting membrane potential (RMP) was not significantly different between AIR-exposed and CIE-exposed neurons, or before and after application of depolarizing current during recording (repeated-measures two-way ANOVA, see Results). ***D***, Inhibition of NBM terminals with 589-nm laser (10 Hz, 60 s) reverses increases in BLA pyramidal neuron firing in CIE neurons (*n* = 6), with no effect in AIR neurons (*n* = 9). Two-way repeated-measures ANOVA, main effect of laser (*p *=* *0.001), trending effect of exposure (*p *=* *0.052), and laser X exposure interaction (*p *=* *0.036). Bonferroni’s *post hoc* tests show significant differences between laser OFF (baseline) and laser ON (**p =* *0.001) and laser ON versus laser OFF (recovery; **p =* *0.003). ***E***, Representative traces of AIR neurons and CIE neurons. Scale bars: *y*-axis 20 mV and *x*-axis20 s for all traces in figure. *Figure Contributions*: Sarah E. Sizer performed the experiments. Sarah E. Sizer analyzed the data.

Next, we sought to understand whether withdrawal-induced potentiation of NBM cholinergic terminal activity modulates BLA pyramidal neuron firing. For these current-clamp recordings, we included picrotoxin in the aCSF to block the influence of NBM GABAergic release. Pilot studies indicated that atropine significantly reduced BLA pyramidal neuron firing independently of AIR or CIE exposure, so it was excluded from the aCSF (data not shown). We injected a depolarizing current into AIR-exposed and CIE-exposed BLA pyramidal neurons to reach a membrane potential of −48 mV and measured action potential firing frequency (Hz) for a 30-s baseline (laser OFF_baseline_), during 60-s laser inhibition of NBM terminals (laser ON), and a 30-s recovery period (laser OFF_recovery_). There was no significant difference in depolarizing current injected in AIR and CIE neurons to maintain –48-mV membrane potential (AIR: 152.7 ± 12.6 pA, *n* = 9 cells from four pairs of rats; CIE: 125.0 ± 22.0 pA, *n* = 6 cells from 3 pairs of rats; unpaired *t* test, *t*_(13)_ = 1.174, *p *=* *0.131; Fig. 6*B*^s^). Also, the resting membrane potential (RMP) was not significantly different between AIR-exposed and CIE-exposed neurons or before and after the application of depolarizing current (repeated-measures two-way ANOVA; no main effect of CIE exposure *F*_(1,10)_ = 0.2158, *p *=* *0.652; no main effect of depolarizing current *F*_(1,10)_ = 3.598, *p *=* *0.087; no CIE X depolarizing current interaction *F*_(1,10)_ = 1.022, *p *=* *0.336; Fig. 6*C*^t^). CIE exposure and withdrawal significantly elevates BLA pyramidal neuron firing frequency at baseline (AIR: 0.3 ± 0.2, *n* = 9 cells from four pairs of rats; CIE: 1.0 ± 0.1, *n* = 6 cells from 3 pairs of rats; planned unpaired *t* test, *t*_(13)_ = 2.679, *p* = 0.0091; Fig. 6*D*^u^). Laser inhibition of NBM terminals reverses enhanced BLA pyramidal neuron firing in CIE neurons, with no effect in AIR neurons (repeated-measures mixed-effects ANOVA; main effect of laser *F*_(2,26)_ = 8.865, *p *=* *0.001; trending main effect of CIE *F*_(1,13)_ = 4.557, *p *=* *0.052; laser X CIE interaction *F*_(2,26)_ = 3.778, *p *=* *0.036; Fig. 6*D*^u^). Bonferroni’s multiple comparison test CIE: OFF_baseline_ versus ON (*t *=* *3.971, *p *=* *0.001) and ON versus OFF_recovery_ (*t *=* *3.725, *p *=* *0.003), AIR: OFF_baseline_ versus ON (*t *=* *0.7082, *p *>* *0.99) and ON versus OFF_recovery_ (*t *=* *1.380, *p *=* *0.540). These data suggest that NBM terminal activity directly contributes to withdrawal-dependent increases in BLA pyramidal neuron firing.

## Discussion

CIE exposure and withdrawal upregulate acetylcholine levels in the BLA (Sizer et al., 2021). Since it is well established that the BLA receives dense cholinergic input from the NBM, we hypothesized that dysregulation of the NBM-BLA circuit may be responsible. Therefore, the present study sought to understand whether alterations in NBM cholinergic neurons and their projections increase glutamatergic signaling in the BLA during ethanol withdrawal. Our data suggest that NBM “cholinergic” neurons respond to synaptic or intrinsic changes following CIE exposure and withdrawal that enhance neuronal excitability. We subsequently used optogenetics to specifically activate or inhibit NBM terminals within the BLA and measure the impact on both stria terminalis glutamate release and BLA pyramidal neuron firing. We found that CIE exposure and withdrawal potentiates NBM cholinergic neurotransmission in the BLA, facilitating tonic activation of nicotinic receptors which helps drive glutamate release from stria terminalis synapses. Upregulation of NBM terminal activity also increases BLA pyramidal neuron firing. Additionally, we found that CIE exposure and withdrawal attenuate NBM GABAergic neurotransmission in the BLA. These findings suggest that withdrawal-induced alterations in NBM afferents may shift the relative impact of cholinergic and GABAergic neurotransmission and contribute to BLA pyramidal neuron excitability. Notably, this is the first study to characterize ethanol-induced alterations of NBM-BLA projections at glutamatergic circuits in the BLA.

**Figure 7.**
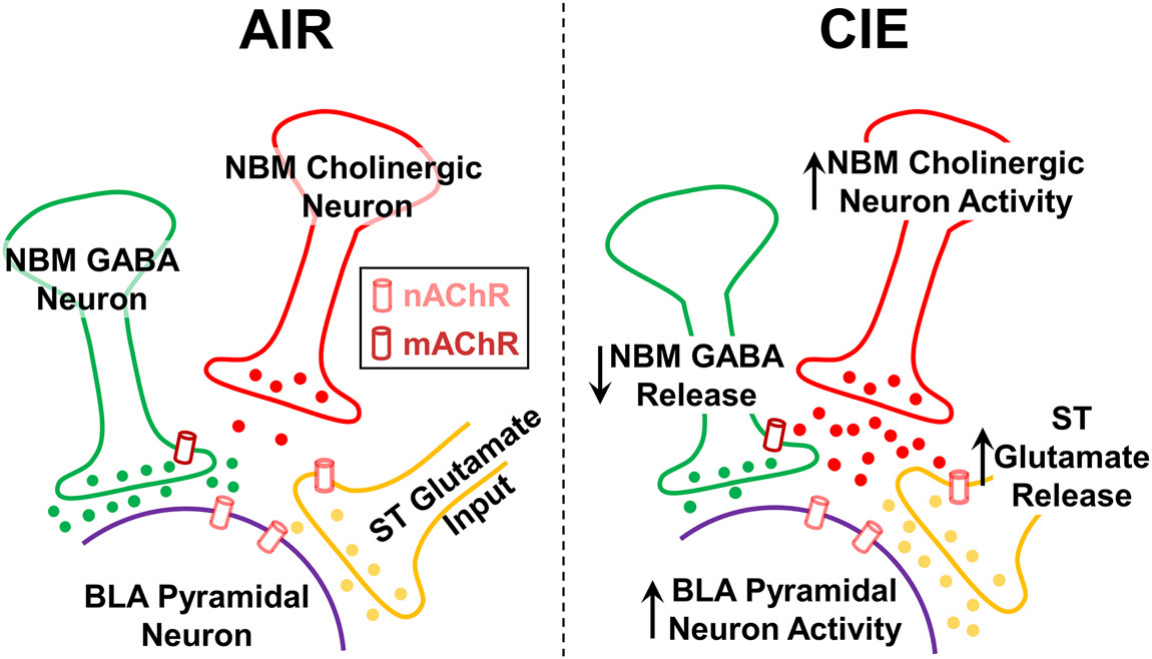
Schematic representation of NBM afferents in AIR control rodents (left) and CIE and withdrawal rodents (right). CIE and withdrawal potentiate NBM cholinergic neuron excitability and increases NBM cholinergic terminal activity in the BLA. The upregulation of NBM acetylcholine release in the BLA tonically activates presynaptic nAChRs and facilitates glutamate release from stria terminalis afferents. CIE also decreases release from NBM GABAergic terminals in the BLA. The effects together appear to enhance BLA pyramidal neuron excitability. *Figure Contributions*: Sarah E. Sizer and Brian McCool composed the figure.

Our initial experiments recorded NBM “cholinergic” and “noncholinergic” neurons in AIR-exposed and CIE-exposed rodents. Consistent with previous reports, we show that the firing frequency of ethanol naive “cholinergic” neurons plateaus at ∼20 Hz (Hedrick and Waters, 2010). Although previous work suggests acute ethanol modulates medial septum/diagonal band cholinergic neuron firing (Ericson et al., 2010), our work appears to be the first study to directly measure the stimulatory effects of chronic ethanol exposure and withdrawal on NBM “cholinergic” neuron excitability. We should note that the variability across these measures may reflect the presence of subpopulations of NBM “cholinergic” neurons. For example, basal forebrain ChAT^+^ cholinergic neurons are characterized as early and late firing neurons that both express distinct firing patterns and may regulate different facets of attention and arousal (Unal et al., 2012). We found that CIE exposure and withdrawal increased the resting membrane potential, increased the action potential peak amplitude, and decreased the half-width of NBM “cholinergic” neurons (Fig. 7). Similar to the effects of ethanol withdrawal, auditory fear conditioning also robustly increases the firing frequency and decreases the half-width of NBM ChAT^+^ neurons (Rajebhosale et al., 2021). Although alterations in the resting membrane potential, peak amplitude, and half-width may be responsible for increases in NBM “cholinergic” neuron excitability, the specific ion channels and signaling pathways underlying these effects warrant further investigation.

However, since synaptic transmission was intact in these experiments, it is impossible to discern whether the alterations in NBM cholinergic neuron action potential properties reflect synaptic influence or intrinsic mechanisms that promote excitability. The extensive list of afferents or circuits which modulate basal forebrain cholinergic neuron excitability makes it difficult to speculate on potential synaptic mechanisms. For example, the NBM receives serotonergic (Gasbarri et al., 1999), noradrenergic, and histaminergic input from the dorsal raphe, locus coeruleus, and hypothalamus, respectively (Zaborszky et al., 2015). BLA-projecting NBM cholinergic neurons also receive input from the striatum [caudate putamen and nucleus accumbens (NAc)] and other basal forebrain regions (Gielow and Zaborszky, 2017). There are three known GABAergic projections from the amygdala to the NBM; these include inputs arising from a small population of intrinsic BLA GABAergic interneurons (McDonald et al., 2012), GABAergic projections from the intercalated cell masses (ICMs; Pare and Smith, 1994; Likhtik et al., 2008), and central amygdala GABAergic projections (Gielow and Zaborszky, 2017). Notably, chronic ethanol reduces GABA release from BLA lateral paracapsular GABAergic cells (Diaz et al., 2011), close relatives of the ICMs. Glutamatergic projections to the NBM also arise from several regions, including the BLA (Arieli et al., 2020), medial PFC (Zaborszky et al., 1997), hypothalamus, and thalamus (Carnes et al., 1990). Dysregulation of any of these neuromodulatory, GABAergic, or glutamatergic afferents could promote NBM cholinergic neuron excitability during ethanol withdrawal. Our study appears to be the first to measure putative NBM cholinergic neuron excitability following CIE exposure/withdrawal and lays the foundation for future work to dissect the specific mechanisms involved.

In order to manipulate NBM terminal activity and measure the neurophysiological outcomes in the BLA, we targeted the NBM through precise stereotaxic microinjection of excitatory and inhibitory AAV viral constructs and found robust GFP expression at both the injection site and the NBM terminals in the BLA (AE Power and McGaugh, 2002; AE Power et al., 2003). Since the NBM-BLA circuit contains cholinergic and GABAergic projections, we used picrotoxin and atropine to interrogate the downstream effects of NBM cholinergic neurotransmission on nAChR-dependent modulation of glutamatergic neurotransmission in the BLA (Jiang and Role, 2008). BLA neurons express both heteromeric and homomeric nicotinic acetylcholine receptors containing different permutations of α_2–4,7_ and β_2,4_ subunits (Zhu et al., 2005). These BLA nicotinic receptors control the expression of anxiety-like and depressive-like behaviors (Mineur et al., 2016; Wise et al., 2020) and regulate fear extinction (Jiang et al., 2016). Importantly, activity of these presynaptic nAChRs can be used as a proxy measure for ethanol-induced alterations in cholinergic signaling at distinct synapses (Sizer et al., 2021). For example, CIE exposure and withdrawal upregulate glutamate release from stria terminalis afferents (Christian et al., 2013; Morales et al., 2018; McGinnis et al., 2020a; Price and McCool, 2022); and this process is driven by the tonic activation of presynaptic α_7_ nAChRs expressed on these glutamate terminals (Sizer et al., 2021). The present study expands on these findings and uses optogenetics to manipulate NBM terminal activity. In AIR controls, optogenetic stimulation of NBM terminals causes nAChR-dependent facilitation of release from stria terminalis inputs. These findings are consistent with recent reports using transgenic rodent models (Tryon et al., 2021) and confirm that optical stimulation of channelrhodopsin-expressing NBM terminals releases acetylcholine. Pharmacological upregulation of synaptic acetylcholine levels produces similar alterations in stria terminalis glutamate release. Since CIE exposure/withdrawal upregulate acetylcholine levels leading to activation of presynaptic nicotinic receptors, optical stimulation of NBM terminals did not further increase glutamate release in this treatment group. However, optical inhibition of these terminals reversed the effect of CIE on stria terminalis glutamate release. Collectively, these data suggest that withdrawal-induced potentiation presynaptic nAChR activity and facilitation of glutamate release relies almost exclusively on NBM cholinergic projections to the BLA (Fig. 7).

Notably, this is also the first study to measure the functional significance of NBM-BLA GABAergic projections. Reports estimate that ∼10% of the NBM-to-BLA projection consists of GABAergic input which may regulate BLA output by inhibiting either “local” GABAergic interneurons or BLA pyramidal neurons (McDonald et al., 2011). The TTX + 4-AP and IPSC latency data strongly suggest that optically-evoked GABAergic responses from NBM terminals are monosynaptic. We cannot however specifically rule out GABA co-release from other NBM projections. Subpopulations of basal forebrain cholinergic neurons express both ChAT and GAD67 mRNA (Sotty et al., 2003); and NBM cholinergic projections co-release GABA in the cortex (Saunders et al., 2015). However, co-localization GABAergic and cholinergic markers in basal forebrain represents ∼1% of the total neuron population (Sotty et al., 2003) suggesting that GABA/acetylcholine co-release may be a relatively rare phenomena. It should be noted that the IPSC amplitude optically evoked from NBM inputs onto BLA principal neurons was significantly larger than the amplitude of NBM excitatory EPSCs. This is consistent with anatomic studies showing that the relative sparse NBM GABAergic terminal fields in the BLA nonetheless contain densely packed synaptic vesicles, numerous mitochondria, multiple synaptic release sites, and large synaptic contact areas (McDonald et al., 2011). Therefore, NBM GABAergic projections may be poised to exert considerable regulatory impact on BLA neurophysiology. Our data also indicate that CIE exposure/withdrawal attenuate NBM GABAergic IPSCs at BLA pyramidal neuron synapses which may reflect the decreased function of these GABAergic projections (Fig. 7). One possible mechanism is that increased cholinergic neurotransmission in the BLA may reduce NBM GABA release via tonic activation of presynaptic M2 muscarinic receptors (Marchi et al., 1990; Salgado et al., 2007; Muller et al., 2016; Fajardo-Serrano et al., 2017). Collectively, our findings illustrate that CIE exposure and withdrawal produce opposing effects on NBM cholinergic and GABAergic neurotransmission onto BLA pyramidal neurons.

Since populations of NBM-BLA cholinergic projection neurons co-express glutamatergic synaptic markers, we found we could optically-evoke EPSCs from NBM terminals in the BLA. The TTX + 4-AP and EPSC latency data demonstrate that these optical synaptic responses likely arise from monosynaptic inputs onto BLA pyramidal neurons. Optically-evoked EPSCs expressed short latencies that were not significantly different between AIR and CIE neurons, yet CIE neurons showed significant increases in the EPSC amplitude across a range of laser intensities. Thus, ethanol withdrawal upregulates direct, monosynaptic NBM excitatory neurotransmission onto BLA principal neurons. Surprisingly, we also found that these optically-evoked EPSCs contain both nicotinic and glutamatergic components. It is unclear whether this reflects acetylcholine/glutamate co-release or optical activation of distinct NBM glutamatergic projections. Approximately one-third of NBM-BLA ChAT^+^ terminals express VGLUT3, suggesting these neurons potentially co-release glutamate (Higley et al., 2011; Nelson et al., 2014). While the substantia innominata-medial PFC circuit contains glutamate-specific projections (Henny and Jones, 2008), NBM glutamatergic projections have not been documented within the NBM-BLA circuit. Regardless, NBM terminals in the BLA express three distinct axonal morphologies which may indicate the presence of a small population of NBM glutamatergic projections (McDonald et al., 2011).

Finally, we used optogenetics to measure the effect of manipulating NBM terminals on BLA pyramidal neuron excitability in AIR-exposed and CIE-exposed groups. Our results suggest that CIE/WD upregulates NBM projections which help promote enhancements in BLA pyramidal neuron firing. This is consistent with recent publications showing that optical stimulation of cholinergic terminals produces sustained excitation of BLA pyramidal neurons (Jiang et al., 2016; Aitta-Aho et al., 2018). While we included atropine in the external aCSF during our synaptic studies (Figs. 3, 5), we did not include atropine in the excitability experiments (Fig. 6) because it alone significantly attenuated BLA pyramidal neuron firing in pilot studies (data not shown). In support of this, muscarinic antagonists occlude long-term potentiation (LTP) in the BLA (Watanabe et al., 1995). Muscarinic receptors could influence BLA pyramidal neuron firing through several mechanisms, including inhibition of the slow-afterhyperpolarization (Washburn and Moises, 1992; Womble and Moises, 1993; JM Power and Sah, 2008) via IP_3_-gated calcium stores (JM Power and Sah, 2007, 2008). These findings suggest that muscarinic receptors play an essential role in regulating BLA pyramidal neuron firing. More broadly, the BLA pyramidal neuron excitability and stria terminalis release studies show that potentiation of NBM cholinergic activity during withdrawal may promote BLA pyramidal neuron excitability through both postsynaptic activation of muscarinic receptors and presynaptic facilitation of glutamate release via nicotinic receptors.

Since male rodents express more robust CIE-dependent changes in BLA glutamate neurotransmission compared with females (Morales et al., 2018; Price and McCool, 2022), we chose to characterize withdrawal-induced dysregulation of the NBM-BLA circuit in male Sprague Dawley rats. One important future direction is clearly to measure NBM-BLA circuit following CIE exposure and withdrawal in females. The NBM is a sexually dimorphic region with respect to the number of ChAT^+^ NBM neurons, ChAT activity, magnitude of acetylcholine release, and density/conductance of presynaptic and postsynaptic cholinergic receptors. These ultimately translate to differential stress reactivity, arousal, and attention between males and females (Mitsushima et al., 2003; Masuda et al., 2005; Takase et al., 2009). Approximately 50–80% of ChAT^+^ neurons in the NBM are immunoreactive for the estrogen receptor GPR30 (Gibbs, 1996; Hammond et al., 2011), and estrogen is protective against cholinergic deficits following chronic stress (Bangasser et al., 2019). Because our studies indicate that NBM cholinergic neurotransmission tightly controls the facilitation of stria terminalis glutamate release during withdrawal (Sizer et al., 2021; Tryon et al., 2021), sex differences within the NBM-BLA circuit may promote disparities related to the effects of CIE exposure at these synapses. Males require fewer days of CIE exposure to express significantly increased stria terminalis glutamate release (Morales et al., 2018). This suggests that BLA neurophysiology may be more vulnerable to CIE exposure in males relative to females. It is therefore reasonable to expect that males could express heightened sensitivity to CIE at NBM cholinergic synapses as well, which may control the differential, sex-specific downstream effects on glutamatergic neurotransmission in the BLA and ultimately related behaviors.

The NBM-BLA circuit strengthens the consolidation of salient cues, a process essential for survival across species. In the presence of an acute stressor, cholinergic NBM neurons fire rapidly (Zhang et al., 2004; Hangya et al., 2015). A variety of acute stressors stimulate NBM cholinergic neuron activity, including mild food shock, predator odor, and exposure to novel/stressful environments (Jing et al., 2020; Rajebhosale et al., 2021; Mineur et al., 2022). NMB cholinergic neuron firing releases acetylcholine within the BLA and modulates attention, arousal, memory storage, reward processing, and threat-encoding (Mark et al., 1996; Jing et al., 2020; Liu et al., 2021). This acetylcholine also strengthens the acquisition of fear-/reward-associated memories, causes deficits in fear extinction (Jiang et al., 2016; Kellis et al., 2020), and increases anxiety-like behavior (Mineur et al., 2018). Importantly, chronic stress causes a persistent elevation in cholinergic signaling that impairs memory encoding, decreases cognitive flexibility, and drives impulsivity and novelty-seeking behaviors (Yilmazer-Hanke et al., 2016; Mineur and Picciotto, 2021). The current work shows that chronic ethanol exposure appears to produce similar persistent changes to NBM cholinergic function. This may contribute to both mood disorders and vulnerability to alcohol (Yilmazer-Hanke et al., 2016).

To conclude, CIE exposure and withdrawal cause neuroadaptations in emotional centers of the brain that facilitate ethanol withdrawal-induced anxiety and relapse. The present study suggests enhancements in NBM “cholinergic” neuron excitability and the downstream effects on BLA neurophysiology are involved in mediating these behaviors. In particular, we show that withdrawal-induced alterations in NBM terminal activity contribute to BLA pyramidal neuron hyperexcitability via three mechanisms. First, ethanol withdrawal strengthens NBM cholinergic neurotransmission and produces tonic activation of nicotinic receptors on stria terminalis glutamatergic synapses, resulting in a “pathologic” elevation of glutamate release. Second, ethanol withdrawal attenuates monosynaptic NBM GABAergic neurotransmission onto BLA pyramidal neurons. Finally, CIE-dependent upregulation of NBM cholinergic terminals helps drive BLA pyramidal neuron excitability during withdrawal. This study thus provides a novel characterization of the NBM-BLA circuit in AIR-exposed and CIE-exposed rodents. Collectively, our results implicate the NBM-BLA circuit in disrupting BLA neurophysiology and potentially mediating anxiety-like behavior during ethanol withdrawal.

## Synthesis

Reviewing Editor: Nathalie Ginovart, Universite de Geneve Decisions are customarily a result of the Reviewing Editor and the peer reviewers coming together and discussing their recommendations until a consensus is reached. When revisions are invited, a fact-based synthesis statement explaining their decision and outlining what is needed to prepare a revision will be listed below. The following reviewer(s) agreed to reveal their identity: Selena Bartlett, Tiffany Wills.

Upon discussion, the reviewers expressed interest in your manuscript and agreed on the significance of the work as a novel contribution to the field and as such support publication. However, both reviewers also believed that revisions could significantly improve the manuscript. Most importantly, since studies were performed in males only, there was a consensus that the inclusion of data in females or a thorough discussion on the inclusion of sex as a biological variable will be essential for improving the manuscript.

There were several other comments and requests for clarifications from both reviewers that should be addressed. Please refer to specific reviewers’ comments below.

Reviewer 1:

The manuscript entitled “chronic intermittent ethanol exposure dysregulates NBM afferents in the BLA” investigates the effect of ethanol on the physiology of putative cholinergic NBM-BLA neurons.

This work is a continuation of a previous study that identified ethanol-induced alterations in cholinergic afferents within the BLA, and now identifies the NBM as the source of these projections, which neurotransmission is altered by ethanol.

The manuscript is very well written, and hypotheses clearly stated, however, providing the author’s names for figure contributions did not follow the anonymity of the rest of the manuscript.

Comments:

1. The manuscript and the field in general, will be greatly advanced by the inclusion of female rodents. The need for improving access to data across all genders is part of a global endeavour to improve the quality of data in scientific research.

2. Although the total number of neurons recorded for each experiment is clearly mentioned, the corresponding number of rats from which these neurons have been recorded is unclear.

3. Brain slice preparation: ketamine 0.1 mM /100 uM appears twice in the brain extraction solution.

4. IHC: 3% PBS is unclear; please provide the concentration of Triton X100 used in PBS-Tx solution

5. Statistics: p<0.0001 denoted as *** is unusual, *** for p<0.001 and **** for p<0.0001 might be less confusing for the reader.

6. Overall, full stops between legends would help the reader.

7. p12. It is mentioned that Fig.1E depicts a “reduction in action potential half-width", however, the p value of p=0.053 suggests that there is no statistical difference.

8. In Fig.1 A, C, D, E, it is not clear why n=11+11 (in A and C) becomes n=10+9 (in D and E). Aren’t the same recordings used across those figures? Same for F-J. If “n=11” appears in Fig.1A, the other “n=X” should also be mentioned.

9. Fig1 legends: there is a problem in the numbering of the representative traces in B and G, for which the arrows are not clearly explained.

10. FIG2. A co-labelling GFP/ChAT could have been useful to appreciate the degree of viral infection. Also, the BLA punctate labelling in Fig2C does not look like afferent labelling. Higher magnifications could therefore be useful in B and C.

11. FIG3. Legends: Problem of numbering from D with Fig3D legend missing. And Fig3G not mentioned in the legend.in the result text it says Fig 5G (p14) instead of Fig. 3B).

12. FIG4D, n= 12 for AIR in legend but only 10 datapoints visible on the figure.

13. FIG5B and 6B, n number missing in legend.

Reviewer 2:

In the current manuscript, “Chronic intermittent ethanol exposure dysregulates nucleus basalis magnocellularis afferents in the basolateral amygdala", the authors evaluate the effects of chronic ethanol on cholinergic neurons in the NBM on BLA transmission. To do this the authors expose male Sprague-Dawley rats to alcohol vapor and withdrawal then recorded from cholinergic and non-cholinergic neurons in the NBM. These cholinergic neurons show increased excitability during ethanol withdrawal which is not seen in non-cholinergic neurons. Exciting or inhibiting these cells in the BLA modulated CIE-induced changes in glutamate release through nAChRs. Inhibiting this projection reduces CIE-induced increases in BLA excitability. The manuscript is well written and contains carefully designed studies that identify a novel circuit changed by chronic ethanol and withdrawal.

Addressing the following points would increase the clarity and context of the current results.

• These studies are performed in male rats only. Clinical data demonstrate that females are more likely to drink/relapse because of stress and negative affect. Further, NIH mandated inclusion or discussion of sex as a factor. Finally, the authors state in the closing paragraph “Male rats exhibit greater sensitivities to the neurophysiological and behavioral effects of CIE and withdrawal than females”. It’s unclear what is being referred to here but also further supports the rational to include sex as a variable.

• While the identification of CIE regulation of this circuit is important, the significance would be increased with a behavioral correlate to this circuit. As generating behavioral data now is probably beyond the scope of this paper, could the authors propose precise phenotypes this circuit might modulate? Specifically, how is this cholinergic projection excited by stimuli and how would that integrate into the expression of withdrawal-induced anxiety

• Non-cholinergic neurons-how were they defined? Are they missing all 3 properties that defined cholinergic neurons? Or is some other designation uses?

• Non-cholinergic neurons had higher variability, could there be multiple populations?

• Summary figure of mechanism

• Paper states that rats were ordered at postnatal days 35-40, what were the ages of surgeries, what were the ages of alcohol treatments.

## References

[B1] Aitta-Aho T, Hay YA, Phillips BU, Saksida LM, Bussey TJ, Paulsen O, Apergis-Schoute J (2018) Basal forebrain and brainstem cholinergic neurons differentially impact amygdala circuits and learning-related behavior. Curr Biol 28:2557–2569.e4. 30100338 10.1016/j.cub.2018.06.064

[B2] Allen TG, Abogadie FC, Brown DA (2006) Simultaneous release of glutamate and acetylcholine from single magnocellular “cholinergic” basal forebrain neurons. J Neurosci 26:1588–1595. 16452682 10.1523/JNEUROSCI.3979-05.2006 PMC6675485

[B3] Alonso A, Khateb A, Fort P, Jones BE, Mühlethaler M (1996) Differential oscillatory properties of cholinergic and noncholinergic nucleus basalis neurons in guinea pig brain slice. Eur J Neurosci 8:169–182. 8713461 10.1111/j.1460-9568.1996.tb01178.x

[B4] Andreasen M, Hablitz JJ (1994) Paired-pulse facilitation in the dentate gyrus: a patch-clamp study in rat hippocampus in vitro. J Neurophysiol 72:326–336. 10.1152/jn.1994.72.1.326 7965017

[B5] Arieli E, Gerbi R, Shein-Idelson M, Moran A (2020) Temporally-precise basolateral amygdala activation is required for the formation of taste memories in gustatory cortex. J Physiol 598:5505–5522. 32857870 10.1113/JP280213

[B6] Ben-Ari Y, Zigmond RE, Shute Cc, Lewis PR (1977) Regional distribution of choline acetyltransferase and acetylcholinesterase within the amygdaloid complex and stria terminalis system. Brain Res 120:435–444. 832133 10.1016/0006-8993(77)90397-3

[B7] Bangasser DA, Eck SR, Ordoñes Sanchez E (2019) Sex differences in stress reactivity in arousal and attention systems. Neuropsychopharmacology 44:129–139. 30022063 10.1038/s41386-018-0137-2 PMC6235989

[B8] Bengtson CP, Osborne PB (2000) Electrophysiological properties of cholinergic and noncholinergic neurons in the ventral pallidal region of the nucleus basalis in rat brain slices. J Neurophysiol 83:2649–2660. 10.1152/jn.2000.83.5.2649 10805665

[B9] Carlsen J, Záborszky L, Heimer L (1985) Cholinergic projections from the basal forebrain to the basolateral amygdaloid complex: a combined retrograde fluorescent and immunohistochemical study. J Comp Neurol 234:155–167. 10.1002/cne.902340203 3886715

[B10] Carnes KM, Fuller TA, Price JL (1990) Sources of presumptive glutamatergic/aspartatergic afferents to the magnocellular basal forebrain in the rat. J Comp Neurol 302:824–852. 10.1002/cne.903020413 1982006

[B11] Christian DT, Alexander NJ, Diaz MR, Mccool BA (2013) Thalamic glutamatergic afferents into the rat basolateral amygdala exhibit increased presynaptic glutamate function following withdrawal from chronic intermittent ethanol. Neuropharmacology 65:134–142. 22982568 10.1016/j.neuropharm.2012.09.004 PMC3521082

[B12] Crouse RB, Kim K, Batchelor HM, Girardi EM, Kamaletdinova R, Chan J, Rajebhosale P, Pittenger ST, Role LW, Talmage DA, Jing M, Li Y, Gao XB, Mineur YS, Picciotto MR (2020) Acetylcholine is released in the basolateral amygdala in response to predictors of reward and enhances the learning of cue-reward contingency. Elife 9:e57335. 10.7554/eLife.57335 32945260 PMC7529459

[B13] Diaz MR, Christian DT, Anderson NJ, Mccool BA (2011) Chronic ethanol and withdrawal differentially modulate basolateral amygdala paracapsular and local GABAergic synapses. J Pharmacol Exp Ther 337:162–170. 21209156 10.1124/jpet.110.177121 PMC3063746

[B14] Dobrunz LE, Stevens CF (1997) Heterogeneity of release probability, facilitation, and depletion at central synapses. Neuron 18:995–1008. 9208866 10.1016/s0896-6273(00)80338-4

[B15] Ericson M, Sama MA, Yeh HH (2010) Acute ethanol exposure elevates muscarinic tone in the septohippocampal system. J Neurophysiol 103:290–296. 19906873 10.1152/jn.91072.2008 PMC2807223

[B16] Fajardo-Serrano A, Liu L, Mott DD, Mcdonald AJ (2017) Evidence for M(2) muscarinic receptor modulation of axon terminals and dendrites in the rodent basolateral amygdala: an ultrastructural and electrophysiological analysis. Neuroscience 357:349–362. 10.1016/j.neuroscience.2017.06.019 28629847 PMC5761332

[B17] Gasbarri A, Sulli A, Pacitti C, Mcgaugh JL (1999) Serotonergic input to cholinergic neurons in the substantia innominata and nucleus basalis magnocellularis in the rat. Neuroscience 91:1129–1142. 10391489 10.1016/s0306-4522(98)00672-1

[B18] Gibbs RB (1996) Expression of estrogen receptor-like immunoreactivity by different subgroups of basal forebrain cholinergic neurons in gonadectomized male and female rats. Brain Res 720:61–68. 10.1016/0006-8993(96)00106-0 8782897

[B19] Gielow MR, Zaborszky L (2017) The input-output relationship of the cholinergic basal forebrain. Cell Rep 18:1817–1830. 28199851 10.1016/j.celrep.2017.01.060 PMC5725195

[B20] Griffith WH, Matthews RT (1986) Electrophysiology of AChE-positive neurons in basal forebrain slices. Neurosci Lett 71:169–174. 3785743 10.1016/0304-3940(86)90553-7

[B210] Gritti I, Henny P, Galloni F, Mainville L, Mariotti M, Jones BE (2006) Stereological estimates of the basal forebrain cell population in the rat, including neurons containing choline acetyltransferase, glutamic acid decarboxylase or phosphate-activated glutaminase and colocalizing vesicular glutamate transporters. Neuroscience 143:1051–1064. 17084984 10.1016/j.neuroscience.2006.09.024 PMC1831828

[B21] Hammond R, Nelson D, Gibbs RB (2011) GPR30 co-localizes with cholinergic neurons in the basal forebrain and enhances potassium-stimulated acetylcholine release in the hippocampus. Psychoneuroendocrinology 36:182–192. 20696528 10.1016/j.psyneuen.2010.07.007 PMC2994977

[B22] Hangya B, Ranade SP, Lorenc M, Kepecs A (2015) Central cholinergic neurons are rapidly recruited by reinforcement feedback. Cell 162:1155–1168. 26317475 10.1016/j.cell.2015.07.057 PMC4833212

[B23] Hedrick T, Waters J (2010) Physiological properties of cholinergic and non-cholinergic magnocellular neurons in acute slices from adult mouse nucleus basalis. PLoS One 5:e11046. 20548784 10.1371/journal.pone.0011046 PMC2883570

[B211] Henny P, Jones BE (2008) Projections from basal forebrain to prefrontal cortex comprise cholinergic, GABAergic and glutamatergic inputs to pyramidal cells or interneurons. Eur J Neurosci 27:654–670 18279318 10.1111/j.1460-9568.2008.06029.x PMC2426826

[B24] Higley MJ, Gittis Ah, Oldenburg IA, Balthasar N, Seal RP, Edwards RH, Lowell BB, Kreitzer AC, Sabatini BL (2011) Cholinergic interneurons mediate fast VGluT3-dependent glutamatergic transmission in the striatum. PLoS One 6:e19155. 21544206 10.1371/journal.pone.0019155 PMC3081336

[B25] Jiang L, Role LW (2008) Facilitation of cortico-amygdala synapses by nicotine: activity-dependent modulation of glutamatergic transmission. J Neurophysiol 99:1988–1999. 10.1152/jn.00933.2007 18272879 PMC2376052

[B26] Jiang L, Kundu S, Lederman JD, Lopez-Hernandez GY, Ballinger EC, Wang S, Talmage DA, Role LW (2016) Cholinergic signaling controls conditioned fear behaviors and enhances plasticity of cortical-amygdala circuits. Neuron 90:1057–1070. 27161525 10.1016/j.neuron.2016.04.028 PMC4891303

[B27] Jing M, et al. (2020) An optimized acetylcholine sensor for monitoring in vivo cholinergic activity. Nat Methods 17:1139–1146. 32989318 10.1038/s41592-020-0953-2 PMC7606762

[B28] Kellis DM, Kaigler KF, Witherspoon E, Fadel JR, Wilson MA (2020) Cholinergic neurotransmission in the basolateral amygdala during cued fear extinction. Neurobiol Stress 13:100279. 10.1016/j.ynstr.2020.100279 33344731 PMC7739185

[B29] Khateb A, Mühlethaler M, Alonso A, Serafin M, Mainville L, Jones BE (1992) Cholinergic nucleus basalis neurons display the capacity for rhythmic bursting activity mediated by low-threshold calcium spikes. Neuroscience 51:489–494. 1488109 10.1016/0306-4522(92)90289-e

[B30] Koob GF, Volkow ND (2016) Neurobiology of addiction: a neurocircuitry analysis. Lancet Psychiatry 3:760–773. 27475769 10.1016/S2215-0366(16)00104-8 PMC6135092

[B31] Lee S, Kim JH (2019) Basal forebrain cholinergic-induced activation of cholecystokinin inhibitory neurons in the basolateral amygdala. Exp Neurobiol 28:320–328. 31308792 10.5607/en.2019.28.3.320 PMC6614066

[B32] Likhtik E, Popa D, Apergis-Schoute J, Fidacaro GA, Pare D (2008) Amygdala intercalated neurons are required for expression of fear extinction. Nature 454:642–645. 18615014 10.1038/nature07167 PMC2528060

[B33] Liu N, Huang K, Wei P, Liu X, Wang L (2021) Modulation of predator cue-evoked tonic immobility by acetylcholine released in the basolateral complex of the amygdala. Neurosci Bull 37:1599–1604. 34478117 10.1007/s12264-021-00767-9 PMC8566626

[B34] López-Hernández GY, Ananth M, Jiang L, Ballinger EC, Talmage DA, Role LW (2017) Electrophysiological properties of basal forebrain cholinergic neurons identified by genetic and optogenetic tagging. J Neurochem 142 [Suppl 2]:103–110. 28791701 10.1111/jnc.14073 PMC7286072

[B35] Marchi M, Sanguineti P, Raiteri M (1990) Muscarinic receptors mediate direct inhibition of GABA release from rat striatal nerve terminals. Neurosci Lett 116:347–351. 2243614 10.1016/0304-3940(90)90099-u

[B36] Mark GP, Rada PV, Shors TJ (1996) Inescapable stress enhances extracellular acetylcholine in the rat hippocampus and prefrontal cortex but not the nucleus accumbens or amygdala. Neuroscience 74:767–774. 10.1016/0306-4522(96)00211-4 8884772

[B37] Mascagni F, Mcdonald AJ (2009) Parvalbumin-immunoreactive neurons and GABAergic neurons of the basal forebrain project to the rat basolateral amygdala. Neuroscience 160:805–812. 10.1016/j.neuroscience.2009.02.077 19285116 PMC2676771

[B38] Masuda J, Mitsushima D, Funabashi T, Kimura F (2005) Sex and housing conditions affect the 24-h acetylcholine release profile in the hippocampus in rats. Neuroscience 132:537–542. 15802204 10.1016/j.neuroscience.2005.01.010

[B39] Mcdonald AJ, Muller JF, Mascagni F (2011) Postsynaptic targets of GABAergic basal forebrain projections to the basolateral amygdala. Neuroscience 183:144–159. 10.1016/j.neuroscience.2011.03.027 21435381 PMC4586026

[B40] Mcdonald AJ, Mascagni F, Zaric V (2012) Subpopulations of somatostatin-immunoreactive non-pyramidal neurons in the amygdala and adjacent external capsule project to the basal forebrain: evidence for the existence of GABAergic projection neurons in the cortical nuclei and basolateral nuclear complex. Front Neural Circuits 6:46. 22837739 10.3389/fncir.2012.00046 PMC3402756

[B41] Mcginnis MM, Parrish BC, Chappell AM, Alexander NJ, Mccool BA (2020a) Chronic ethanol differentially modulates glutamate release from dorsal and ventral prefrontal cortical inputs onto rat basolateral amygdala principal neurons. eNeuro 7:ENEURO.0132-19.2019. 10.1523/ENEURO.0132-19.2019 PMC7070451 31548367

[B42] Mcginnis MM, Parrish BC, Mccool BA (2020b) Withdrawal from chronic ethanol exposure increases postsynaptic glutamate function of insular cortex projections to the rat basolateral amygdala. Neuropharmacology 172:108129–108129. 10.1016/j.neuropharm.2020.108129 32418906 PMC7313316

[B43] Mineur YS, Picciotto MR (2021) The role of acetylcholine in negative encoding bias: too much of a good thing? Eur J Neurosci 53:114–125. 31821620 10.1111/ejn.14641 PMC7282966

[B44] Mineur YS, Fote GM, Blakeman S, Cahuzac EL, Newbold SA, Picciotto MR (2016) Multiple nicotinic acetylcholine receptor subtypes in the mouse amygdala regulate affective behaviors and response to social stress. Neuropsychopharmacology 41:1579–1587. 26471256 10.1038/npp.2015.316 PMC4832019

[B45] Mineur YS, Cahuzac EL, Mose TN, Bentham MP, Plantenga ME, Thompson DC, Picciotto MR (2018) Interaction between noradrenergic and cholinergic signaling in amygdala regulates anxiety- and depression-related behaviors in mice. Neuropsychopharmacology 43:2118–2125. 29472646 10.1038/s41386-018-0024-x PMC6098039

[B46] Mineur YS, Mose TN, Vanopdenbosch L, Etherington IM, Ogbejesi C, Islam A, Pineda CM, Crouse RB, Zhou W, Thompson DC, Bentham MP, Picciotto MR (2022) Hippocampal acetylcholine modulates stress-related behaviors independent of specific cholinergic inputs. Mol Psychiatry 27:1829–1838. 10.1038/s41380-021-01404-7 34997190 PMC9106825

[B47] Mitsushima D, Masuda J, Kimura F (2003) Sex differences in the stress-induced release of acetylcholine in the hippocampus and corticosterone from the adrenal cortex in rats. Neuroendocrinology 78:234–240. 10.1159/000073707 14583656

[B48] Morales M, Mcginnis MM, Robinson SL, Chappell AM, Mccool BA (2018) Chronic intermittent ethanol exposure modulation of glutamatergic neurotransmission in rat lateral/basolateral amygdala is duration-, input-, and sex-dependent. Neuroscience 371:277–287. 29237566 10.1016/j.neuroscience.2017.12.005 PMC5809207

[B49] Muller JF, Mascagni F, Zaric V, Mott DD, Mcdonald AJ (2016) Localization of the M2 muscarinic cholinergic receptor in dendrites, cholinergic terminals, and noncholinergic terminals in the rat basolateral amygdala: an ultrastructural analysis. J Comp Neurol 524:2400–2417. 26779591 10.1002/cne.23959 PMC5094197

[B50] Nelson AB, Bussert TG, Kreitzer AC, Seal RP (2014) Striatal cholinergic neurotransmission requires VGLUT3. J Neurosci 34:8772–8777. 24966377 10.1523/JNEUROSCI.0901-14.2014 PMC4069355

[B51] Nickerson Poulin A, Guerci A, El Mestikawy S, Semba K (2006) Vesicular glutamate transporter 3 immunoreactivity is present in cholinergic basal forebrain neurons projecting to the basolateral amygdala in rat. J Comp Neurol 498:690–711. 10.1002/cne.21081 16917846

[B52] Pare D, Smith Y (1994) GABAergic projection from the intercalated cell masses of the amygdala to the basal forebrain in cats. J Comp Neurol 344:33–49. 10.1002/cne.903440104 7520456

[B53] Pidoplichko VI, Prager EM, Aroniadou-Anderjaska V, Braga MF (2013) alpha7-Containing nicotinic acetylcholine receptors on interneurons of the basolateral amygdala and their role in the regulation of the network excitability. J Neurophysiol 110:2358–2369. 10.1152/jn.01030.2012 24004528 PMC3841870

[B54] Power AE, Mcgaugh JL (2002) Cholinergic activation of the basolateral amygdala regulates unlearned freezing behavior in rats. Behav Brain Res 134:307–315. 12191818 10.1016/s0166-4328(02)00046-3

[B55] Power AE, Mcintyre CK, Litmanovich A, Mcgaugh JL (2003) Cholinergic modulation of memory in the basolateral amygdala involves activation of both m1 and m2 receptors. Behav Pharmacol 14:207–213. 12799522 10.1097/00008877-200305000-00004

[B56] Power AE, Thal LJ, Mcgaugh JL (2002) Lesions of the nucleus basalis magnocellularis induced by 192 IgG-saporin block memory enhancement with posttraining norepinephrine in the basolateral amygdala. Proc Natl Acad Sci U S A 99:2315–2319. 11830635 10.1073/pnas.022627799 PMC122362

[B57] Power JM, Sah P (2007) Distribution of IP3-mediated calcium responses and their role in nuclear signalling in rat basolateral amygdala neurons. J Physiol 580:835–857. 17303640 10.1113/jphysiol.2006.125062 PMC2075466

[B58] Power JM, Sah P (2008) Competition between calcium-activated K+ channels determines cholinergic action on firing properties of basolateral amygdala projection neurons. J Neurosci 28:3209–3220. 18354024 10.1523/JNEUROSCI.4310-07.2008 PMC6670694

[B59] Price ME, Mccool BA (2022) Chronic alcohol dysregulates glutamatergic function in the basolateral amygdala in a projection- and sex-specific manner. Front Cell Neurosci 16:857550. 35496915 10.3389/fncel.2022.857550 PMC9050109

[B60] Rajebhosale P, Ananth M, Crouse R, Jiang L, Hernández GL, Arty C, Wang S, Jone A, Zhong C, Desai NS, Li Y, Picciotto MR, Role LW, Talmage DA (2021) Basal forebrain cholinergic neurons are part of the threat memory engram. bioRxiv. 10.1101/2021.05.02.442364 PMC10928508 38363713

[B61] Ray JP, Price JL (1992) The organization of the thalamocortical connections of the mediodorsal thalamic nucleus in the rat, related to the ventral forebrain-prefrontal cortex topography. J Comp Neurol 323:167–197. 10.1002/cne.903230204 1401255

[B62] Salgado H, Bellay T, Nichols JA, Bose M, Martinolich L, Perrotti L, Atzori M (2007) Muscarinic M2 and M1 receptors reduce GABA release by Ca2+ channel modulation through activation of PI3K/Ca2+ -independent and PLC/Ca2+ -dependent PKC. J Neurophysiol 98:952–965. 10.1152/jn.00060.2007 17581851

[B63] Saunders A, Granger AJ, Sabatini BL (2015) Corelease of acetylcholine and GABA from cholinergic forebrain neurons. Elife 4:e06412. 10.7554/eLife.06412 PMC4371381 25723967

[B64] Sim JA, Allen TG (1998) Morphological and membrane properties of rat magnocellular basal forebrain neurons maintained in culture. J Neurophysiol 80:1653–1669. 9772229 10.1152/jn.1998.80.4.1653

[B65] Sizer SE, Parrish BC, Mccool BA (2021) Chronic ethanol exposure potentiates cholinergic neurotransmission in the basolateral amygdala. Neuroscience 455:165–176. 33385490 10.1016/j.neuroscience.2020.12.014 PMC7856184

[B66] Sliedrecht W, De Waart R, Witkiewitz K, Roozen HG (2019) Alcohol use disorder relapse factors: a systematic review. Psychiatry Res 278:97–115. 31174033 10.1016/j.psychres.2019.05.038

[B67] Sotty F, Danik M, Manseau F, Laplante F, Quirion R, Williams S (2003) Distinct electrophysiological properties of glutamatergic, cholinergic and GABAergic rat septohippocampal neurons: novel implications for hippocampal rhythmicity. J Physiol 551:927–943. 10.1113/jphysiol.2003.046847 12865506 PMC2343277

[B68] Takase K, Kimura F, Yagami T, Mitsushima D (2009) Sex-specific 24-h acetylcholine release profile in the medial prefrontal cortex: simultaneous measurement of spontaneous locomotor activity in behaving rats. Neuroscience 159:7–15. 10.1016/j.neuroscience.2008.12.039 19162130

[B69] Tryon SC, Bratsch-Prince JX, Warren JW, Jones GC, Mcdonald AJ, Mott DD (2021) Differential regulation of prelimbic and thalamic transmission to the basolateral amygdala by acetylcholine receptors. bioRxiv 2021.12.28.474396. 10.1523/JNEUROSCI.2545-21.2022 PMC9899087 36535767

[B70] Unal CT, Golowasch JP, Zaborszky L (2012) Adult mouse basal forebrain harbors two distinct cholinergic populations defined by their electrophysiology. Front Behav Neurosci 6:21. 22586380 10.3389/fnbeh.2012.00021 PMC3346982

[B71] Unal CT, Pare D, Zaborszky L (2015) Impact of basal forebrain cholinergic inputs on basolateral amygdala neurons. J Neurosci 35:853–863. 25589777 10.1523/JNEUROSCI.2706-14.2015 PMC4293427

[B72] Washburn MS, Moises HC (1992) Muscarinic responses of rat basolateral amygdaloid neurons recorded in vitro. J Physiol 449:121–154. 1522506 10.1113/jphysiol.1992.sp019078 PMC1176071

[B73] Watanabe Y, Ikegaya Y, Saito H, Abe K (1995) Roles of GABAA, NMDA and muscarinic receptors in induction of long-term potentiation in the medial and lateral amygdala in vitro. Neurosci Res 21:317–322. 10.1016/0168-0102(94)00867-F 7777222

[B74] Williams S, Serafin M, Mühlethaler M, Bernheim L (1997) Distinct contributions of high- and low-voltage-activated calcium currents to afterhyperpolarizations in cholinergic nucleus basalis neurons of the guinea pig. J Neurosci 17:7307–7315. 10.1523/JNEUROSCI.17-19-07307.1997 9295377 PMC6573441

[B75] Wise T, Patrick F, Meyer N, Mazibuko N, Oates AE, Van Der Bijl AHM, Danjou P, O’Connor SM, Doolin E, Wooldridge C, Rathjen D, Macare C, Williams SCR, Perkins A, Young AH (2020) Cholinergic modulation of disorder-relevant neural circuits in generalized anxiety disorder. Biol Psychiatry 87:908–915. 32107005 10.1016/j.biopsych.2019.12.013 PMC7198974

[B76] Womble MD, Moises HC (1993) Muscarinic modulation of conductances underlying the afterhyperpolarization in neurons of the rat basolateral amygdala. Brain Res 621:87–96. 8221077 10.1016/0006-8993(93)90301-3

[B77] Woolf NJ, Butcher LL (1982) Cholinergic projections to the basolateral amygdala: a combined Evans Blue and acetylcholinesterase analysis. Brain Res Bull 8:751–763. 10.1016/0361-9230(82)90102-2 6182963

[B78] Yilmazer-Hanke D, Eliava M, Hanke J, Schwegler H, Asan E (2016) Density of acetylcholine esterase (AchE) and tyrosine hydroxylase (TH) containing fibers in the amygdala of roman high- and low-avoidance rats. Neurosci Lett 632:114–118. 10.1016/j.neulet.2016.08.053 27585749

[B79] Zaborszky L, Gaykema RP, Swanson DJ, Cullinan WE (1997) Cortical input to the basal forebrain. Neuroscience 79:1051–1078. 10.1016/S0306-4522(97)00049-3 9219967

[B80] Zaborszky L, Csordas A, Mosca K, Kim J, Gielow Mr, Vadasz C, Nadasdy Z (2015) Neurons in the basal forebrain project to the cortex in a complex topographic organization that reflects corticocortical connectivity patterns: an experimental study based on retrograde tracing and 3D reconstruction. Cereb Cortex 25:118–137. 10.1093/cercor/bht210 23964066 PMC4259277

[B81] Zhang YQ, Lü SG, Ji YP, Zhao ZQ, Mei J (2004) Electrophysiological and pharmacological properties of nucleus basalis magnocellularis neurons in rats. Acta Pharmacol Sin 25:161–170. 14769203

[B82] Zhu PJ, Stewart RR, Mcintosh JM, Weight FF (2005) Activation of nicotinic acetylcholine receptors increases the frequency of spontaneous GABAergic IPSCs in rat basolateral amygdala neurons. J Neurophysiol 94:3081–3091. 16033935 10.1152/jn.00974.2004

[B83] Zwart R, Vijverberg HP (1997) Potentiation and inhibition of neuronal nicotinic receptors by atropine: competitive and noncompetitive effects. Mol Pharmacol 52:886–895. 9351980 10.1124/mol.52.5.886

